# What’s in it for citizen scientists? An analysis of participant’s gains from a democratisation perspective

**DOI:** 10.12688/openreseurope.17436.2

**Published:** 2024-10-14

**Authors:** Elisabeth Unterfrauner, Claudia Magdalena Fabian, Gary Hemming, Beatriz Garcia

**Affiliations:** 1 Zentrum fur Soziale Innovation, Vienna, Vienna, Austria; 2 Osservatorio Gravitazionale Europeo, Cascina, Tuscany, Italy; 3 Consejo Nacional de Investigaciones Cientificas y Tecnicas, Mendoza, Argentina

**Keywords:** Citizen science, evaluation, pre-post-design, diversity, inclusion

## Abstract

Citizen science projects optimise the democratisation of the production of scientific knowledge. In these initiatives, research processes do not rely solely on scientists’ but on citizens’ engagement likewise with benefits on both sides. As previous work shows, the democratisation perspective of citizen science projects might be viewed critically as some groups of citizens tend to be overrepresented in these initiatives while other are left out. This paper explores the claim of democratisation and the citizens’ benefits based on four citizen science projects in the fields of astrophysics and particle physics on the citizen science platform Zooniverse. Besides a general engagement strategy, the citizen science projects addressed two groups specifically, the elderly and people with visual impairments. The claim for democratisation is reflected in the analysis of citizens’ demographic variables as an indicator for accessibility of the research projects. We used a pre-post design with questionnaires on science attitudes, motivations, skills, self-efficacy, and knowledge to assess what citizen scientists gained from participating in the project. The demographic analysis of the data reveals that participants were quite heterogeneous and that people who feel that they belong to a group that is discriminated against are particularly motivated to participate in citizen science projects. In terms of benefits, the results indicate knowledge and scientific skills gains, but no changes on other evaluative dimensions. Their attitude towards science was, in general, already rather positive when joining the projects, thus not leaving much room for change. These results confirm the importance of and call for a diversified citizen science engagement strategy and show that even in citizen science projects where the citizens’ task is limited to classifying data lead to scientific knowledge and skills gains.

## Introduction

Citizen science, defined as collaborative research with a varying degree of involvement of citizens in scientific processes (c.f. Heigl *et al*., 2019), is not a recent phenomenon. Even if it was not known by the name ‘citizen science’ in the 19th century, aspects of the approach can be found in earlier forms of collaboration between scientists and lay people. For instance, the Christmas Bird Count, initiated by the National Audubon Society in 1900, is recognised as one of the oldest and most notable citizen science projects ( Dunn *et al*., 2005). It involved volunteers documenting bird species and populations during the winter season. With growing environmental awareness, citizen science projects focusing on the monitoring of pollution and ecological changes began to emerge. Notable examples include the Cornell Lab of Ornithology's Breeding Bird Survey (started in 1966) and the Community Collaborative Rain, Hail, and Snow Network (CoCoRaHS) established in 1998. The advent of the internet and digital technologies subsequently revolutionised citizen science. Online platforms, such as Zooniverse, launched in 2007, allowed volunteers to contribute to various research projects through the analysis of large datasets and images.

What is new in the rise of the modern form of citizen science is a more radical involvement of volunteers in the scientific process, questioning the traditional relationship between scientific knowledge production and its reception (e.g. Delfanti, 2010). The idea of citizen science holds the idealistic promise to bridge the gap between scientists and citizens, with benefits on both sides ( Robinson *et al*., 2018). While the role of lay people was merely limited to assisting in the collection of data in early collaborations, the degree of involvement of volunteers in current citizen science projects varies, with their inclusion in different phases throughout the research process.

Citizen science is about democratisation of access to science ( Curtis, 2018; Giardullo *et al*., 2023; Peters & Besley, 2019), breaking up the so-called ‘ivory tower’ of science, and an empowerment of citizens in the scientific undertaking ( Herzog & Lepenies, 2022). Researchers implementing citizen science projects acknowledge how involving citizens brings in different perspectives and that some of the responsibilities and duties in the research process are being shared ( Shirk & Bonney, 2018).

The participation of citizen scientists ranges from active engagement in scientific activities and processes, to contributions to evidence-based policy evaluation and development ( Haklay, 2015; Rowland, 2012).

**Figure 1.**
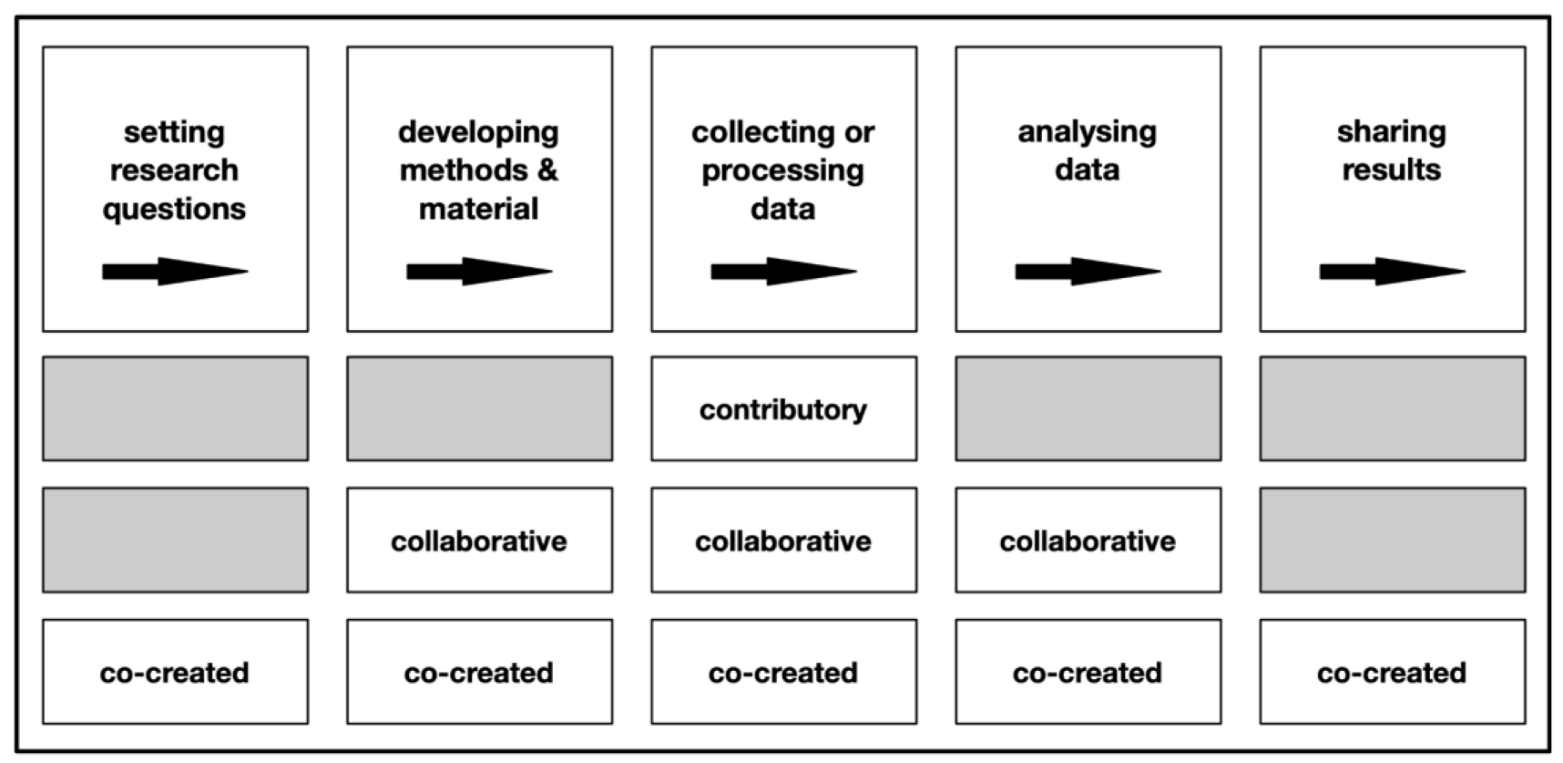
Stages of the scientific process non-scientist participants are involved in according to the type of citizen science project, adapted from West and Pateman ( 2017, cited after Reynolds *et al.,* 2021).

Levels of involvement and engagement vary, depending upon the type of citizen science project and the stage of the research process (c.f. Figure 1).

According to the so-called ‘extreme citizen science’ approach ( Chiaravalloti *et al*., 2022), citizens should be involved in all phases of the scientific process, from co-defining research questions to disseminating results. People with different educational backgrounds should be able to access and participate in citizen science projects so that a true democratisation of science can take place. ‘Extreme’ here refers to the extent of participation and to people who were previously excluded from the production of scientific knowledge. The ‘extreme citizen science’ approach calls for inclusive projects that are accessible regardless of background, counteracting the pattern of ‘white, well-educated and male participants’ in citizen science projects ( Curtis, 2018).

Thus, citizen science projects require open scientific practices and a positive attitude towards citizen science by the researchers. However, one can pose the question, whether democratisation of knowledge production through citizen science projects does succeed, implying that people are equally represented irrespective of their genders, ethnicity, disability, etc., and whether traditional boundaries between researchers and lay people are broken up. Furthermore, we can ask whether the motivation to join and the potential benefits differ between marginalised and non-marginalised people. 

One of the most prominent motivational drivers for citizen scientists, according to a number of reviewed citizen science projects, is the desire to contribute to a “greater good” and to help science to solve problems that are perceived as relevant and meaningful in today’s society ( Land-Zandstra *et al*., 2016; Martin *et al*., 2016; Silva *et al*., 2016). Once citizen scientists are active and involved, intrinsic motivators and social influences gain more importance in keeping them active and engaged ( Nov *et al.*, 2011) and in increasing not only the quantity, but also the quality of their contributions ( Nov *et al*., 2014). However, as motivations differ over time and also between different groups of people, the need to study distinct motivations per group become apparent in order to understand socio-cultural interests and barriers to join ( Ryan *et al.*, 2001). Our understanding of who volunteers in scientific endeavors are and what motivates them to participate remains limited ( Woosnam *et al.*, 2019).

### Accessibility and inclusiveness of citizen science projects

For citizen science projects to hold true to the goal of democratisation, people with diverse backgrounds have to be represented among citizen scientists reflecting the demographics of the general population. Multiple perspectives from all parts of society add value to scientific discourses and allow for a multi-faceted interpretation of scientific results avoiding blind spots and biases. Bonney *et al.* (2016) conclude that citizen science should strive to include a diverse range of people to contribute to the democratisation of science. 

Whether citizen science initiatives do persist inequalities between more privileged and less privileged people has been investigated in previous studies relying on demographic data of participants, in terms of age, gender, educational background, etc. However, a full picture of who participates in citizen science projects is missing ( Paleco *et al.*, 2021). As studies indicate, an equal representation of the population in citizen science projects investigated along the lines of demographic data, has not been achieved with some groups of people being overrepresented. In the US for in instance, Pandya *et al.* (2018), found that individuals from historically unrepresented groups participated less often than majority groups in citizen science initiatives. The same accounts for people with disabilities in the UK, whose participation does not represent the share of people with disabilities in the overall population (OPAL report, cited after Paleco *et al.*, 2021). This trend of underrepresentation of certain groups does not only apply to citizen science projects but to participative research in general such as environmental volunteering as earlies studies show (e.g. Ockenden, 2007)

How to increase the accessibility of citizen science projects, strategies to promote inclusion are being discussed and researched by groups such as the European Citizen Science Association (ECSA) with its working group on Empowerment, inclusiveness, and equity ^1^.

### Assessing benefits for citizen scientists

Although appreciation for citizen science projects continues to grow, the number of projects demonstrating the impact on involved citizen scientists and diverse demographic characteristics is still limited. Outcomes of the collaboration between citizen science and research can be manifold, depending on the type of project (e.g. Bonney *et al*., 2009) and the involvement of citizens at different stages of the project. As such, the evaluation of project goals and outcomes can also be manifold. Up to now, by far the most commonly investigated scientific outcomes of citizen science initiatives concentrate upon the number of related scientific publications. Another aspect that can be observed in various types of citizen science projects is the development of new skills and knowledge by citizen scientists (e.g. Schäfer *et al.*, 2020; Wiggins & Crowston, 2015). Firstly, there are references to the importance of knowledge gains related to the research topic as being the most important impact for participants ( Stepenuck & Green, 2015). Secondly, the involvement in citizen science activities teaches the participants about the process of scientific enquiry and helps them to gain a deeper understanding of scientific outcomes ( Bela *et al*., 2016; Richter *et al*., 2016; Riesch & Potter, 2014). The citizen science approach inspires stewardship, and enhances the sense of participant empowerment ( Crall, 2010; Crall *et al*., 2013; Sutcliffe, 2011; Wickson & Carew, 2014).

Experts recommend defining specific goals, expected learning outcomes, and a customised evaluation strategy with measurable indicators ( Jordan *et al*., 2012; Phillips *et al*., 2014). For the evaluation strategy, the pre-existing knowledge and skills of the target groups must be aligned with the expected learning outcomes, in order to be able to properly assess participant learning gains and assess the impact of the project ( Skrip, 2015).

Citizen scientists have been directly approached to assess what they felt to be the benefits of their participation in projects and to measure their learning outcomes. Cox *et al*. (2015) differentiate between contribution to science and public engagement. Others have released guidelines on how to set-up a citizen-science project, including recommendations regarding their evaluation, such as Bonney *et al*. (2009), who suggest the evaluation of scientific literacy outcomes through the use of similar indicators, such as the duration of involvement by project participants; the numbers of participant visits to the project website; but also direct surveys directed at citizens, in order to measure how understanding of science content and of science processes improves, etc. A study on measuring outcomes in citizen science projects, Bonney *et al*. (2016), found, through surveys of citizen-science practitioners and additional interviews, the following constructs to be achievable and measurable: interest in science and nature; self-efficacy for science and environmental action; motivation for science and environmental action; science enquiry skills; data interpretation skills; knowledge of the nature of science; and environmental stewardship. To support the evaluation of citizen science projects, the Cornell Lab of Ornithology elaborated evaluation guidelines that focus especially on learning outcomes, such as the acquisition of new knowledge and skills, but also on increased interest in science, motivation, self-efficacy in science-participation, personal development and behavioural change ( Phillips *et al*., 2014).

The most frequently used evaluation instruments are not only survey interviews, and the analysis of participant communication ( Gommerman & Monroe, 2012), but also stakeholder consultations, observations, iterative adaptations with actors in the field and self-assessment tools applied during the evaluation process ( Kieslinger *et al*., 2017).

- While previous studies have provided valuable insights into the evaluation of citizen science projects, they often rely on diverse methods that may not comprehensively capture the full range of participant outcomes, especially when self-reported data is involved ( Peter *et al*., 2019). In our study, we build on these foundational works but seek to address some of their limitations by focusing on a more robust, multi-dimensional approach. Specifically, we aim to investigate the democratisation of access to astrophysics and particle physics citizen science projects and explore how these opportunities, along with individual learning gains, vary across demographic variables. This leads us to the following research questions: (i) What do citizen scientists gain in terms of attitude towards science, motivation to join, self-efficacy, scientific skills, and knowledge acquisition in astrophysics and particle physics, in the four citizen science projects?
- (ii) Are there differences in terms of motivation to join and gains between different groups in respect to their genders, age, educational background, and experience of discrimination?

The first research question refers to the overall sample to measure the effects of participation in the four citizen science projects in general, while the second specifically is meant to reveal whether the aim of democratisation has been achieved, both in terms of equal representation of diverse populations and their gains.

While evaluation studies describing the benefits for citizen scientists do exist (as cited above), the analysis from a democratisation perspective has largely been limited to counting participant numbers across groups ( Hecker *et al.*, 2018), with little further investigation into their individual gains and motivations. In the following sections Next, we describe the methods used to gather evidence of participants’ personal benefits, followed by an overview of the overall results. Finally, in the discussion and conclusion section, we address the two research questions and identify potential gaps for future research.

## Four citizen science projects on Zooniverse

The four citizen science projects were developed as part of the REINFORCE project, which was funded within the Science With And For Society theme of the EU Horizon 2020 framework and were implemented on the Zooniverse platform ( https://www.zooniverse.org/). On Zooniverse, volunteers interested in participating in research can contribute online to different projects across a broad range of research areas. The platform also encourages citizens to engage in dialogue with research teams on dedicated discussion forums, known as ‘Talk-pages’ to address open questions and concerns.

The four citizen science projects can be classified as a mix of contributory and collaborative, rather than co-creative citizen science projects (c.f. Figure 1) as citizen scientists do not collect data but analyse already collected data shared by large research infrastructure. According to Shirk and Bonney (2018) the projects on the Zooniverse platform are a prime example of the “ *technology transfer”* category, where citizens can classify digital images and contribute to citizen science projects. As the exercises to be done mainly involved categorisation tasks, opportunities were provided to interact with researchers and other citizen scientists and to ‘dive deeper’ in the respective fields of research. These comprised online interaction options (online forum, webinars, online visits to research infrastructure) as well as face-to-face events (public lectures, course for seniors, artistic interventions).

**Figure 2.**
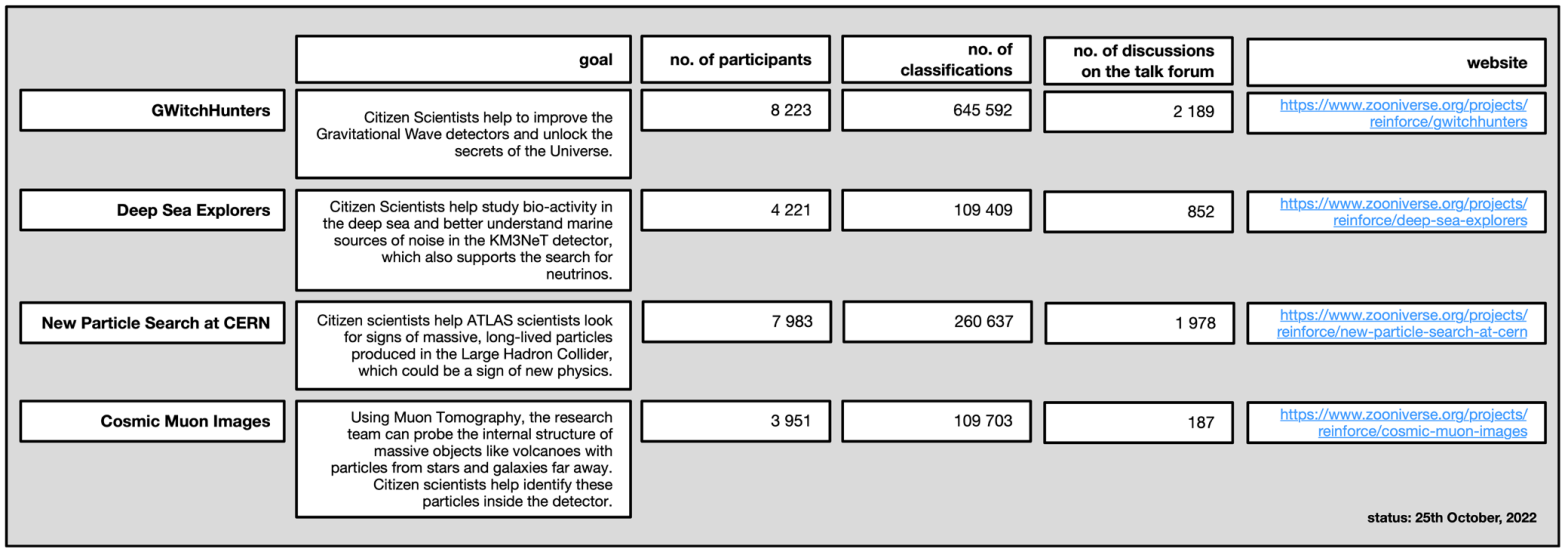
Overview of the four citizen science projects.

The REINFORCE implementation period of the four citizen science projects ran from the 19th of October 2021 to the 25th of October 2022, although all four of them are still available online (overview in Figure 2).

### GWitchHunters

The GWitchHunters ^2^ project developed an advanced citizen science programme by providing access to representations of gravitational wave (GW) data produced by the Virgo ^3^ detector. These included data taken from the GW strain channel, as well as from auxiliary channels, providing environmental background information. Since the sensitivity of GW detectors is limited by several types of noises, it is crucial to understand their origin and impact on data acquired. By ‘hunting’ for noises and systematically profiling them, the research team can undertake in-depth analyses and contribute to the development of a more efficient detector with a wider detection span. The citizen scientists in GWitchHunters contributed to this activity by looking at chunks of data and identifying transient noise artefacts, known as ‘glitches.’ The outcome of these activities was used to train machine learning algorithms to automatically recognise and isolate these glitches in GW detector data.

The GWitchHunters research team also collaborated with a sister project, GravitySpy ^4^; also available on Zooniverse, which is a highly successful citizen science project developed using data from the LIGO ^5^ detectors, based in the United States.

### Deep Sea Explorers

In the Deep Sea Explorers ^6^ project on Zooniverse, citizen scientists helped to optimise the categorisation of data gathered by the KM3NeT neutrino telescope, which collects environmental noise from the bottom of the deep sea in two locations: one to the south of France; the other off the coast of Sicily, Italy, in the Mediterranean Sea. By participating in the analysis on Zooniverse, citizen scientists were able to engage with the world of neutrino astronomy and gain at the same time insights into the unexplored deep marine environment. In the framework of the Deep Sea Explorers project, citizen scientists were invited to classify different sources of bioluminescence, recorded by the detector, and bioacoustic signals registered by hydrophones situated in the surrounding environment.

### New Particle Search at CERN

The New Particle Search project at CERN ^7^ engaged citizen scientists with data recorded by the ATLAS detector of the Large Hadron Collider (LHC) at CERN. For the purpose of the New Particle Search project, the researchers developed a specific software for the display and analysis of the ATLAS data (HYPATIA ^8^) and asked the citizen scientists to look for evidence of undiscovered particles. On the platform, the citizen scientists were able to classify static images, interact with the event display, select specific tracks, and calculate invariant masses. Some particle decays, such as photon conversion, were more accurately identified by humans than by algorithms and, with the aggregated data from thousands of citizen scientists, the researchers had the possibility to explore and examine their data further. The data categorised by citizen scientists can be compared to the categorisations produced by machine learning algorithms and can serve as a baseline for further research. The citizen scientists received valuable feedback from the New Particle Search researchers and were able to draw their attention to interesting events for further investigation.

### Cosmic Muon Images

The Cosmic Muon Images ^9^ project focuses on interdisciplinary studies involving geoscience and archaeology and aimed to show how technology can be used to study fundamental physics and develop frameworks that have a significant impact on society. In the project, researchers provided citizen scientists with an open data set, recorded by cosmic-ray detectors during a period of data-taking at the Apollonia Tumulus in Greece, in 2018, and invited them to interact with the data and make classifications.

## Inclusion strategy

To reach diverse audiences and engage them as REINFORECE engagement strategy included specific approaches for elderly people and people with visual impairments, groups that are often hard to reach in online citizen science projects due to the need for digital access and content accessibility. Digital infrastructures can constitute both an opportunity and an obstacle for reaching more diverse groups ( Paleco *et al.*, 2021). However, data on participation of people with visual impairments in online citizen science projects are lacking to date. 

There are two solutions to make citizen science projects more diverse; (i) to strive for more representative representation of certain groups and (ii) to make the content and the exercises of the citizen science projects more inclusive. While the first one can be achieved with a tailored engagement strategy, the second aim lies with the researchers preparing the citizen science initiative with the description of tasks and communication to potential participants. During pre-testing of the alpha version with laypeople and through iteration and feedback loops with the Zooniverse team, the four citizen science projects were fine-tuned to maximise accessibility. Simple language was used omitting too specific terms and the tutorials for the citizen science projects were designed in a way to make them concise with concrete explanations how to perform the categorisation tasks with a couple of trial exercises.

Two of the four projects were translated into additional languages for reducing language barriers (GWitchHunters: English and Italian; New Particle Search at CERN: English, Greek and Spanish).

The goal of the citizen science projects was clearly communicated as well as the potential contribution of citizen scientists to the success of the projects. A concise explanation of the role of involved scientists, along with contact persons and a forum for interaction, was provided to facilitate communication between citizen scientists and the research team, as well as among citizen scientists themselves, who gradually formed a community and offered each other advise when needed. 

Additionally to these general inclusion strategies, the inclusion strategy for elderly included a series of designed courses and for visually impaired individuals, making the digital images accessible through sound. 

### Inclusion strategy for elderly citizen scientists via specifically designed courses

As part of the REINFORCE project, a course on science specifically for senior citizens, was organised in collaboration with the organisation *Università della Libera Età* (University of the Free Age), based near to the Virgo gravitational-wave detector, in Cascina, near Pisa. The course was structured around approximately monthly sessions, given in-person, at the home of the University group: the municipal library of Cascina. The calendar for the meeting had to be reformulated on more than one occasion on account of the Covid-19 pandemic making it necessary to push sessions back, while the pandemic also contributed to fluctuations in attendance over the life cycle of the implementation of the course. The first implementation closed with a visit by more than 40 members of the group to the European Gravitational Observatory (EGO), the home of the Virgo detector, and led to a second edition of the course being implemented over the following academic year, well beyond the natural lifetime of the REINFORCE project itself. The course implementation covered the following areas: Classical particle physics; particles & waves in the XX century; waves: concept and detection; the cosmology of the (in)visible Universe; citizen science: from theory to practice; general relativity; brainstorming and resolution of technical and theoretical problems; the sonification of gravitational waves; and art & science.

Course sessions were delivered by professors and researchers from the University of Pisa and members of the REINFORCE collaboration based at EGO. Most of the sessions were delivered in Italian, as most of the group were mother tongue Italian speakers and did not speak a second language. Despite this, two of the sessions - those dedicated to the cosmology of the (in)visible Universe and that on art and science - were delivered in English, were both very well received by participants, leading to the conclusion that, where sessions material was potentially more accessible, especially when the themes covered were supported with visually explicative presentation, it was better suited to cut through and hold participant attention even when delivered in a different language.

The overall evaluation session carried out face-to-face with participants and separately with session providers, proved fruitful and provided several useful suggestions. It was clear that participants had initially felt somewhat daunted by the syllabus that had been prepared and were concerned that the bar had been set too high. It was also clear that a standard lecture scenario was also not necessarily particularly helpful in developing an environment that was conducive to the group feeling at ease and comfortable. The evaluation session made it possible to re-engineer subsequent sessions to allow for more give-and-take between session providers and participants. For example, simply removing the lectern from the session and locating the provider more closely to the group, made the process more natural and more fluid. The group grew to become more informal and, as a consequence, more lively, which was ultimately more beneficial for all. For elderly however it was particularly important to receive training on the digital infrastructure used, i.e. the Zooniverse platform.

### Inclusion strategy for citizen scientists with visual impairments through sonification of data

Increasing the senses, increasing inclusion, was the “big argument” for the development of a software tool - *sonoUno* - dedicated to data sonification. The ambition of this work is to expand the senses used in scientific inference, beyond the visual, and to include in the general effort of the scientific community, people with sensory disabilities (especially visual).

In sciences such as astrophysics and physics, scientists constantly interact with numerical data, generally represented visually. These interactions imply a response that is mostly related to current events, and which are limited by the data analysis tools available and the resolution of display elements such as screens. Studies ( Diaz Merced, 2013) show that multisensory display of data can improve signal detection, especially if it is astronomical data. This allows us to infer that a sound recording alongside its visualisation can contribute to a better understanding of results, and, as such, allow people with functional diversity to analyse scientific data, and then contribute to scientific discoveries.

Since 1962, 98 sonification projects had been developed ( Zanella *et al.* (2022)); however many of them were discontinued, lacked documentation, or had no evidence of applications in science. Not all of them share the same objective: some are tools to produce sound through the command line, others have the purpose of offering the user the ability to modify the sound configurations to achieve a sound system that fits their needs, and others prioritise the development of an accessible graphical interface. In this sense, sonoUno is a pioneering software designed with the primary goal of enabling citizens to engage in research through a multisensory approach, allowing them to analyse the data scientifically. A first training workshop in data detection by sound (performed in August 2022) allowed us to obtain important results, as well as to confirm earlier ones, and evaluate the performance of a group with the new tool. It has become evident that the possibility to use sound improves the integration of people with disabilities in the study of science and the multimodal approach helps the understanding of conceptual and scientific content, allowing the same phenomenon to be explored through different sensory channels, for disabled and non-disabled people alike. In a closing survey, interesting results emerged, such us the recognition of the technique, and comments such as “I think we will use it in my next work” or,” the multisensorial analysis can improve my own work”.

After the first encounter with citizen participating in the citizen science projects, the sonoUno team prepared a series of training activities, and invited these participants to test them, with a very good response. The user-centred design approach, which ensured ongoing feedback from users, also contributed to the development of better training activities and ultimately led to the creation of a new training platform ( https://sonotraining.um.edu.ar/). Through this platform, we are collecting data on the perception and effectiveness of sonification for data analysis from both blind and sighted users, as well as trained and untrained people.

The acceptance of this technique among professionals is growing, as evidenced by new workshops, conferences, and invited talks in international events, as well as the inclusion of data analysis using sound in professional papers ( Fovino *et al.*, 2024; Trayford & Harrison, 2023; Trayford *et al.*, 2023; Tucker Brown *et al.*, 2022) as a complement to traditional techniques. se two strategies were developed in addition to the general engagement strategy of the project with dedicated seminars, tours and live visits to the research infrastructures, outreach via social media channels and the projects websites and arts-based approaches The aim was to offer further formats for interaction beyond the digital representation of the four projects on the Zooniverse project and the forum tool to interact digitally. Due to the Corona pandemic, some of the events had to be moved online. For instance, an originally planned tour on the VIRGO premises had to be transformed into a virtual tour. Unfortunately, re unable to determine how many event participants or recipients of additional offerings subsequently registered on the Zooniverse platform. Although this information was requested in the survey, the relevant section was left incomplete in the majority of responses.

## Methods and materials

In our study, in collaboration with the research teams, we discussed potential gains in the four astrophysics and particle physic domains, following Skrip’s (2015) suggestion that learning outcomes should be clearly defined in advance. To structure the different evaluative dimensions, we relied on the logic model by Kurz and Kubek (2016), which although not specifically designed for citizen science projects, is a useful instrument to differentiate between outputs, outcomes, and impacts (cf. Table 1).

**Table 1.** Logic model of Reinforce citizen science projects.

1 Outputs (what we do)	2 Outcomes (results at target group level)	3 Impact (results at societal level)
**1a Output** • Web-based interface (Zooniverse) • Sonification • Citizen education (citizen training activities) e.g. vision building workshop, online and in-situ training, practice reflection, etc. • Community empowerment and awareness activities e.g. workshops, summer/ winter school, Science café, open schooling day, etc. • Educational resources	**2a New knowledge, skills,** **attitudes and awareness** Citizens Scientist: • new knowledge: comprehend role of large RI; understanding basic physics concepts; principles of machine learning; methods of scientific investigations • skills: data recognition and analysis skills, critical thinking • attitude: awareness of science/scientific work (e.g. collaboration, daily scientific processes); inclusiveness of science; increasing awareness of the interaction with nature; identification with their direct contribution to science Researcher: new knowledge: further research insights; gaining more experiences with citizen science projects and their potential for future CS projects	**3 Social and economic ** **impact** • Enhance science literacy of the society, public understanding of science, critical thinking • Economic costs and benefits of citizen science • Enablers and barriers for development of new knowledge citizen’s science Science career motivation
**1b Use of output by target groups** • Participation in Zooniverse projects • Participation in Citizen education • Participation in Community empowerment activities Reach of different target groups (elderly, visual impairments, pupils)	**2b Change actions/behaviour** • Science career motivation • Cooperation with researchers ○ Collaboration between citizen scientists and researchers ○ Experience exchange among citizen scientists Improving mutual understanding through exchanging diverse expertise on a larger scale	
**1c Participants satisfaction** • Zooniverse project experience • F2F event (Citizen education; community empowerment)	**2c Living conditions** • Citizens feel empowered by contributing to science • Participation is possible even in confinement (e.g. Covid-19)	

According to the logic model, outputs are what the projects offer, its use and the participants’ satisfaction; outcomes are what the project aims to achieve with a target group; and impacts are the contributions of the project on a societal level. A general logic model, applicable to all four citizen science projects, was developed in collaboration with the research teams. This model included an additional layer of customisation tailored to the specific fields of research, forming the foundation for the subsequent operationalization of various dimensions. To measure the outcomes on the individual participant level, a one-group pre-test/post-test design ( Levine & Parkinson, 2014) was implemented. This design falls under quasi-experimental designs as the main premise of true experiments, namely the existence of a control or comparison group and the random selection and assignment of participants, is missing. As a result, although one would be able to assume that changes from the pre-test to the post-test are due to the participation of citizen scientists in the citizen science projects, unlike in true experiments, in which such effects would be solely attributed to this participation; in this design, outside factors cannot be controlled or ruled out. Nevertheless, this design is more reliable and provides more accurate data than a one-group post-only design, which, due to the lack of a pre-test, cannot show any change in relation to skills, knowledge, attitudes, behaviours, level of awareness, etc.

### Constructing the pre/post questionnaire

**Figure 3.**
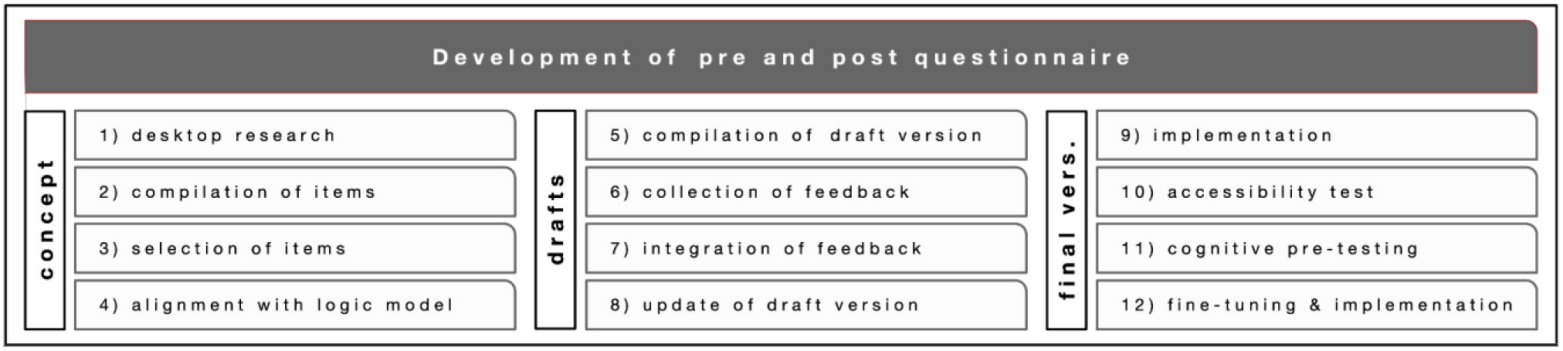
Development of the pre- and post-questionnaire.

The development of the questionnaire involved the following steps (see overview in Figure 3): (1) desk research on evaluation surveys in citizen science projects with a similar focus; (2) a compilation of items from different already available surveys that were suitable for our purposes; (3) first selection of items; (4) alignment with the general logic model and the specific logic models of each of the four citizen science projects, respectively; (5) compilation of a draft version; (6) collection of feedback by research teams and user testing with 10 volunteers; (7) integration of feedback; (8) update of another draft version; (9) implementation on the online survey tool LimeSurvey ^10^; (10) accessibility check by people with visual impairments; (11) cognitive pre-testing ( Prüfer & Rexroth, 2005) with potential users and ‘thinking aloud’ protocols to test the usability aspects of the items and detect potential misunderstandings; (12) last fine-tuning of the questionnaire and integrating the link to the survey in the four Zooniverse projects.

Desk research on citizen science studies (c.f. step 1) focused on the evaluation of citizen science projects and Zooniverse projects in particular, and their evaluation instruments and questionnaires, respectively. As far as our desk research indicates, only rarely has a pre-post design been used in Zooniverse projects. In their survey on Planet Hunters ( Depper, 2019), another Zooniverse project, citizen scientists' learning gains were assessed retrospectively, through a question on whether volunteers felt they had learned anything and, if so, what they had learned. This post-only design obviously has several flaws; not least individual memory capacity, which might influence the results ( Medici *et al*., 2010).

The User’s Guide for Measuring Learning Outcomes in Citizen Science ( Phillips *et al*., 2014) released by the Cornell lab for Ornithology, has become one of the standard resources in the quest to assess citizen science project outcomes on a participant level, including several questionnaires that have already been widely used and which have been checked in terms of their quality, such as reliability and validity ( Phillips *et al*., 2018). Some of these questionnaires have been framed as general questionnaires, which can be used mostly for any kind of citizen science project, and custom questionnaires, which can be adapted to the specific context. Thus, the questionnaires constitute a valid source for our study. All questionnaires were screened and those items that were in line with the logic model (c.f. step 3 and 4 in the development of our questionnaire) were extracted. Other additional questions resulted from “inspiration” from the other evaluation studies cited above and from the interaction with, and feedback provided by, project partners and demonstrator research teams and the citizen science expert team (c.f. steps 5, 7 and 9).

In the pre/post-test design as applied in our study, the pre-survey aims to measure the “baseline.” In our case, this covered five distinct areas: knowledge (self-reported and tested knowledge), motivation to join, self-efficacy, skills, and attitudes. Each of these dimensions comprises five and 19 items (cf. *Extended data,* complete questionnaire; Unterfrauner & Fabian, 2024).

Thus, eventual changes in these areas could be assessed in comparison with the post-survey. Although the four citizen science projects were slightly different in nature, they each required the performance of broadly similar tasks, i.e. the classification of data representations. The field of research differed, however, and this was reflected in the construction of the questionnaire knowledge items, where the single items differed slightly across the four citizen science projects.

The questionnaires were implemented in LimeSurvey, and the collected data imported for further analysis in the statistical analysis software SPSS.

The link to the pre-questionnaire was included in the Zooniverse project tutorials. Participants were required to go through the tutorials to understand how to classify data and contribute to the individual projects. Participants were asked to share their email addresses when they first filled in the questionnaire and received an email with a link to the post-questionnaire one month after filling in the pre-questionnaire. The timeframe for sending out the post-questionnaire after one month was chosen as appropriate because experiences from other Zooniverse projects suggested that most classifications per participant do not extend over this period. Also, other studies refer to this timeframe to measure active engagement ( Strasser *et al.*, 2023).

For technical reasons it was not possible to include the post-questionnaire on Zooniverse with impact on response numbers (see below).

The data collection using the surveys for each of the projects started when they were each launched as official projects on Zooniverse. New Particle Search at CERN became an official Zooniverse project on the 26th of October 2021; GWitchHunters on the 16th of November, 2021; Cosmic Muon Images on the 11th of January, 2022; and Deep Sea Explorers on the 8th of February, 2022. The data collection period ended on the 17th of August 2022.

## Results

Analyses of participant data showed that there were no significant differences in overall proportion between responses from the four projects. Consequently, the four sets of responses were merged into an overall dataset for further analysis ( Unterfrauner, 2024). The limitations of the data lie in the fact that, as with all voluntary participation, the dataset is limited to only those people who took the time to complete the questionnaire and thus represents a self-selected sample ( Bethlehem, 2010). The data might therefore not be fully representative of all participants in the four projects. Nevertheless, they shed light on the demographics, gains in knowledge, skills, and changes in attitude towards science and allow for a differentiated analysis of changes, taking into account demographic aspects and experiences of discrimination.

The analysis of the demographic characteristics of the citizens participating in the four projects is based on the data of the pre-questionnaire, in order to give a more comprehensive picture, while, for the comparison of pre-questionnaire and post-questionnaire scores, it was possible to use only complete datasets, i.e. where the respondent had completed both questionnaires.

While the pre-questionnaire was filled in by a total of 1,179 participants, the post questionnaire had only 301 responses, resulting in a response rate of 25.5% in the second round. 

### Demographic characteristics

**Figure 4.**
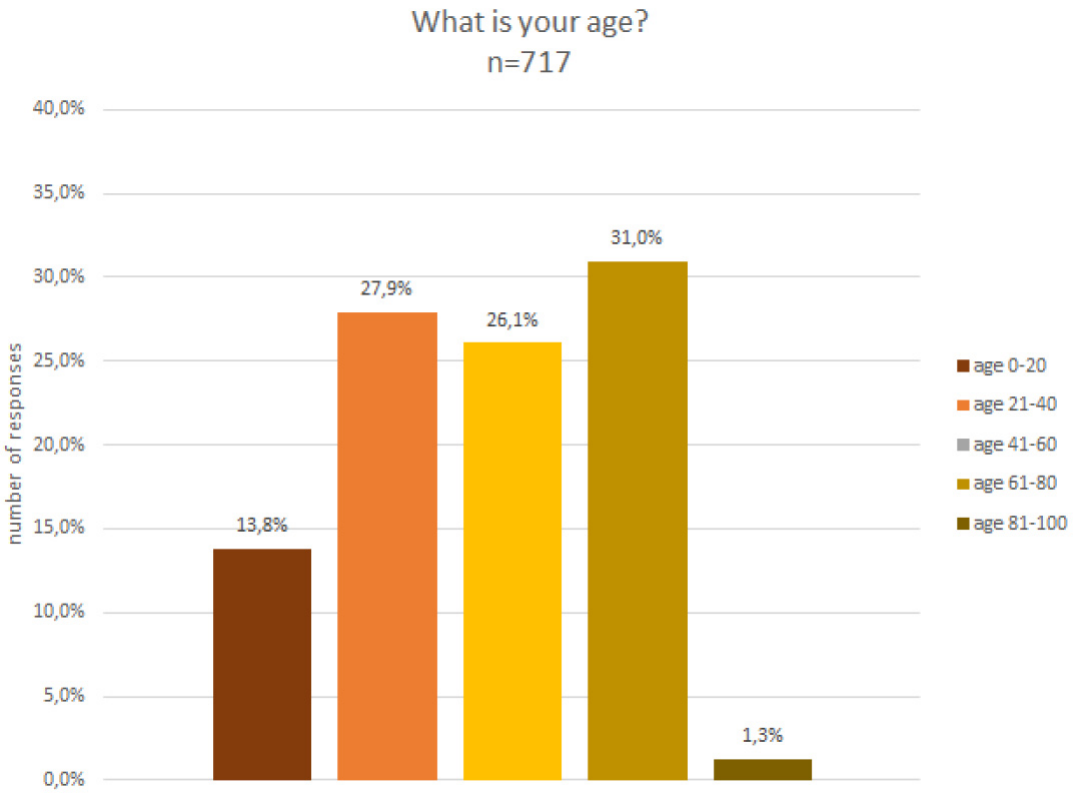
Demographic data - age.

**Figure 5.**
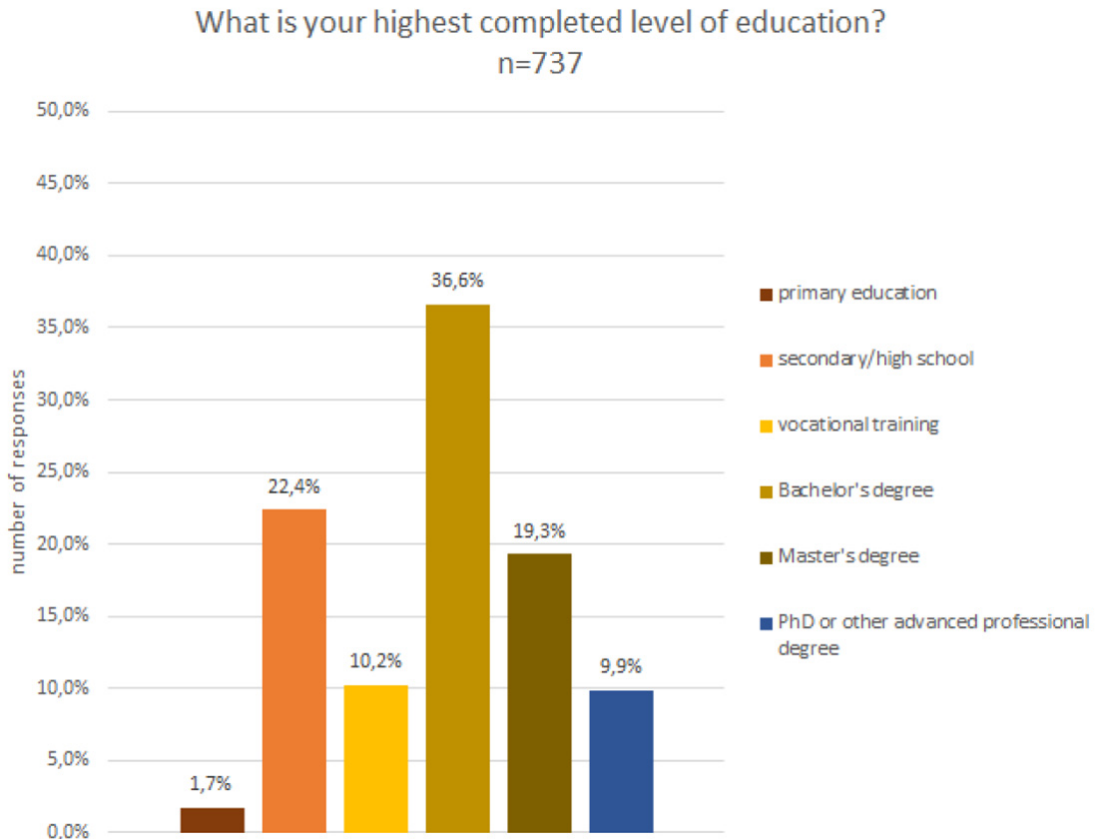
Demographic data - education.

The analysis of the gender composition indicates that 53% were male, 40% female, 3% preferred not to say and 4% defined themselves as non-binary. In terms of age (c.f. Figure 4) and educational level (cf. Figure 5), participants were quite diverse. About 13% were below the age of 20 and a small fraction, i.e. 1%, were above the age of 80, while the remaining age classes, between 20 and 80 years old, were fairly equally represented in the sample.

A closer look at the composition regarding educational level reveals that the sample is skewed towards higher education degrees, including Bachelor’s, Master’s and PhDs or other advanced professional degrees. However, also people with lower educational degrees accessed the projects and were able to contribute. More than one third of the participants had not completed an academic degree.

More than half indicated that they had no professional background in science (52%), 38% had a scientific background and 8% were not totally sure.

**Figure 6.**
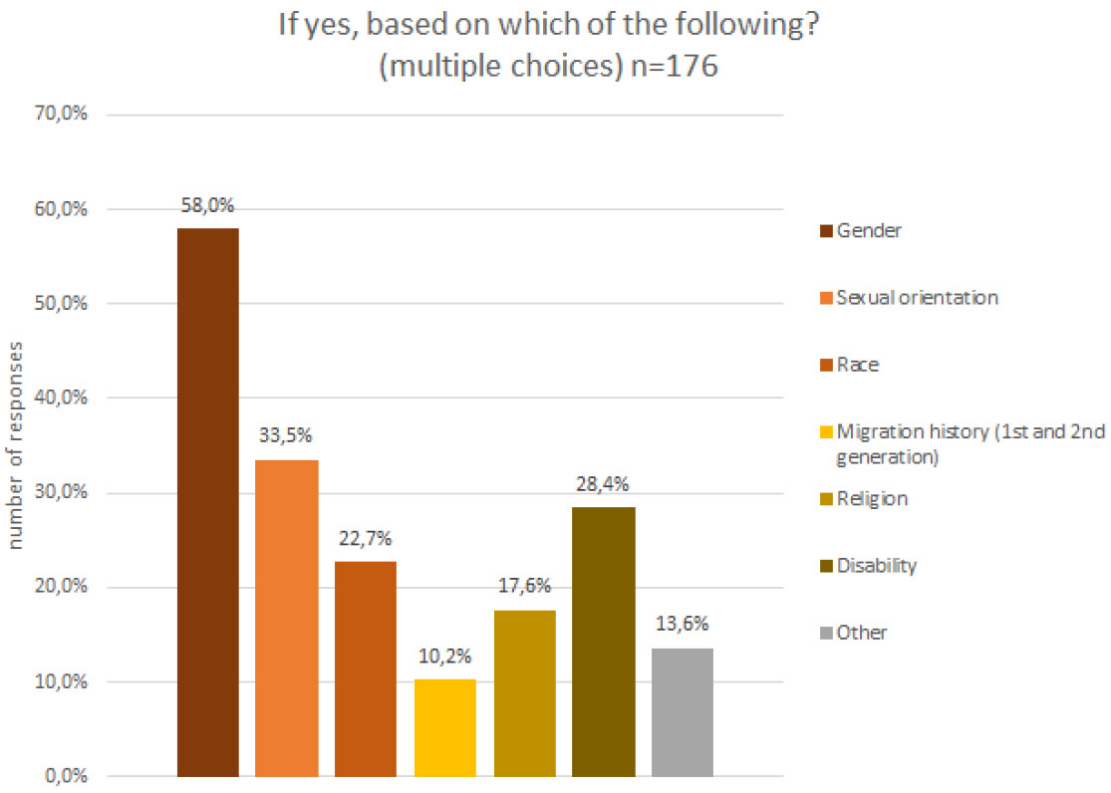
Demographic data – discrimination experiences.

A few participants (5%) indicated a visual impairment, which required assistive technology and could not be compensated for with glasses, and about a quarter of all participants indicated that they felt they belonged to a discriminated against group. The discrimination experience is attributed to multiple different factors (c.f. Figure 6), the majority feeling discriminated against for their gender identity and sexual orientation, followed by disability, ethnic group and migration history.

### Engagement level

On average, the respondents made 109 classifications (with a standard deviation of 826 and a maximum of 24,179 (!!) classifications) and spent 2.09 hours on the project (standard deviation of 14.8 hours and a maximum of 160 hours) ^11^. The average classification time was 60 seconds (with a standard deviation of 112 sec and a max of 595 sec).

The level of engagement in terms of performed classifications and time spent on the Zooniverse projects varied to a great extent. Thus, engagement levels are also taken into account in the analysis of changes.

### Changes in motivation, attitudes, knowledge, self-efficacy, and skills

In the following, we describe the changes resulting from the comparison between pre and post-test scores on the dimensions: attitude, motivation to join, scientific skills, self-efficacy in relation to scientific undertaking, reported scientific knowledge and tested scientific knowledge.

The pre-questionnaire served to measure the ‘baseline,’ i.e. scores before participating in the citizen science projects, to compare with the scores from post-questionnaires, which were filled in after one month of participating in the citizen science projects.

The following table shows the results of the Wilcoxon -tests. The Wilcoxon signed-rank test is a non-parametric statistical test used to compare two related samples or repeated measurements on a single sample to assess whether their population mean ranks differ or as in our case before and after participating in the citizen science projects. For the comparison of groups, Mann-Whitney U-tests were applied. The Mann-Whitney U-test is a non-parametric statistical test used to compare differences between two independent groups when the dependent variable is either ordinal or continuous but not normally distributed.

The first five subscales and resulting scores are based on a 5-point Likert scale, from 1=strongly disagree to 5=strongly agree. In the last subscale on tested knowledge there were three answer options, i.e. ‘yes,’ ‘no,’ and ‘don’t know.’ The score on tested knowledge can range between 0 and 1, indicating the ratio of correct answers.

**Figure 7.**
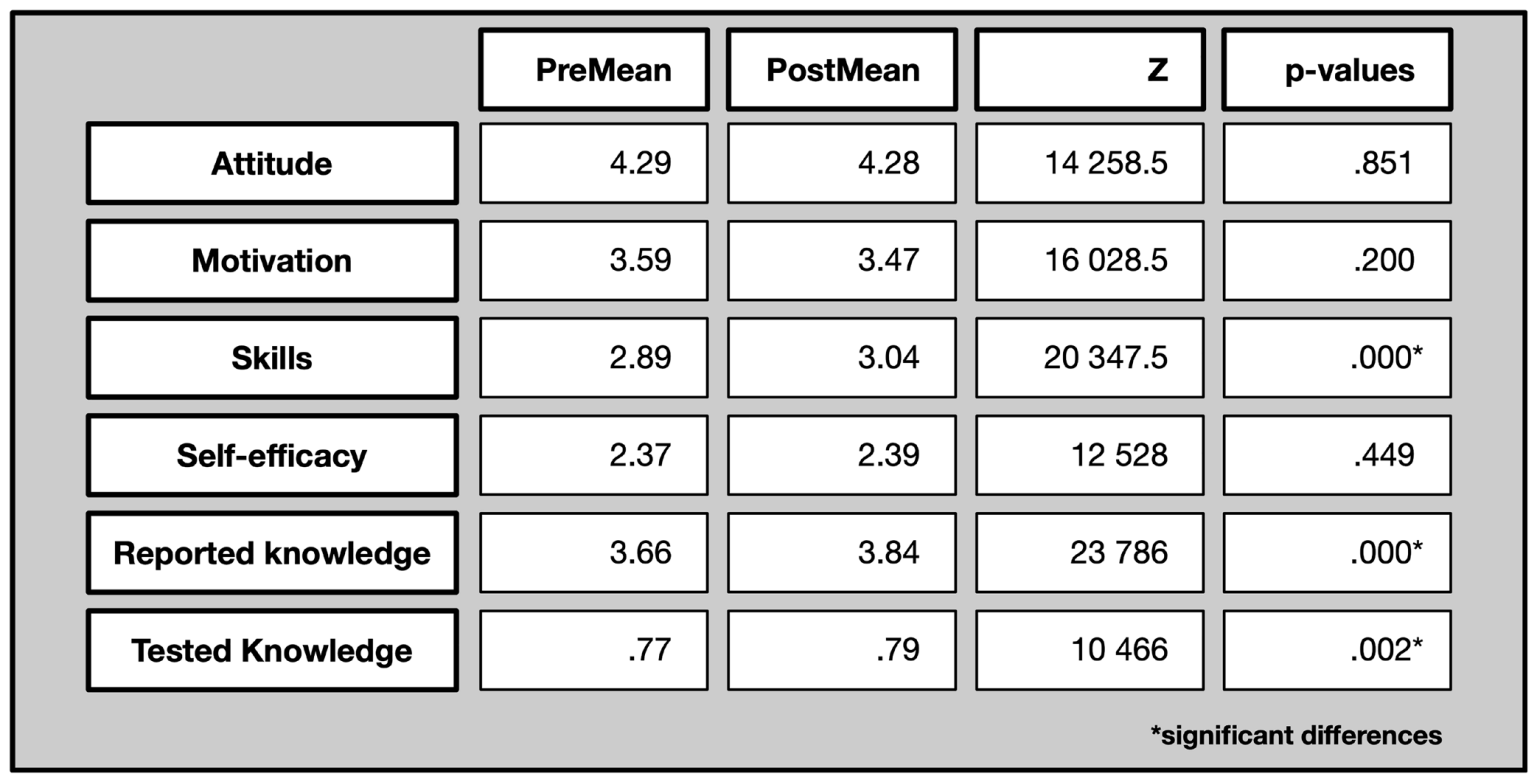
Pre and post comparison on different evaluative dimensions.

The following overview in Figure 7 shows the average scores (means) per evaluation dimension, both in the pre- and the post-survey (first two columns: PreMean and PostMean) as well as results from the Wilcoxon test for assessing significant differences. (last column).

Across different evaluation dimension, both significant and non-significant differences were observed. In detail, it is particularly scientific skills, reported and tested knowledge where participants increase their scores. Their attitude towards science, their motivation to join, and their perceived self-efficacy does not change significantly.

In the following we will investigate more details on subscale level.

*Attitude towards science:* This subscale comprises nine items, with statements such as “I am interested in learning more about particle physics” or “I enjoy reading about science related topics” (See complete questionnaire in annex).

The attitude towards science in general was already rather high in the beginning (with an average mean of 4.29) and did not change over the course of participation.

*Motivation to join:* This subscale consists of twelve items and comprises items covering intrinsic and extrinsic motivation. Intrinsic motivation describes an inherent satisfaction for a certain activity, while extrinsic motivation describes a behaviour determined by external rewards or punishments ( Ryan & Deci, 2000). Example of items are: “Because I think it’s a good thing to do” (intrinsic), “Because I believe in can contribute to scientific research” (extrinsic) and “For the recognition I get from others” (extrinsic).

The motivation to join is on average on a medium level with a score of 3.58, which remains at the same level. A detailed item-level analysis indicates that this is primarily due to r intrinsic rather than extrinsic motivation. For instance, participants chose to join because they wanted to spend their spare time doing something useful (item 3b.3, see Annex), or because they enjoyed getting involved in scientific activities (item 3b.4), both reflecting intrinsic motivation. In contrast, fewer participants were motivated by gaining recognition from others (item 3.9) or by connecting with their professional activities (item 3b.6), which are extrinsic motivation factors.

*Science Skills:* The question block on science skills comprised five statements referring to the citizen science project (e.g. ‘I know how to categorise the data in the Deep Sea Explorers project”). As the Wilcoxon-test shows, there is a significant increase in science skills from the pre- to the post-survey. In other words, participants, according to their own ratings, gained scientific skills over the course of their participation in the Zooniverse projects.

*Self-efficacy:* Four items related to self-efficacy in doing science, i.e. believing in one’s own skills in science-related activities, included statements such as ‘I think I am pretty good at following instructions for scientific activities’ and ‘It takes me a long time to understand how to do scientific activities’. Perceived self-efficacy in science-related skills did not improve significantly and remained at a medium level across both time points, with scores averaging around 2.3.

*Knowledge:* The knowledge block was divided into reported knowledge and tested knowledge, which allowed for a comparison of an objective and subjective level of knowledge in the respective field. In the reported knowledge section (nine items), participants were asked whether they felt confident explaining specific scientific terms, while in the tested knowledge part they had to identify which items were correct and which were incorrect. These ‘objective’ items comprised statements regarding the purpose of research infrastructures and some statements referring to the specific scientific fields of the individual citizen science project. Participants, both subjectively and objectively, improved their knowledge in the field.

### Demographic analysis of score changes

In the following, the pre- and post-test results are contrasted against the demographic characteristics of the participants, allowing for an analysis from a democratisation perspective.

*Gender differences*: Due to the small proportion of people who declared themselves as non-binary and people who preferred not to say (c.f. demographic analysis), the data associated with these were omitted from the following analysis.

No significant differences were found between females and males in their Zooniverse engagement, in terms of number of classifications, time spent per classification, and overall time.

**Figure 8.**
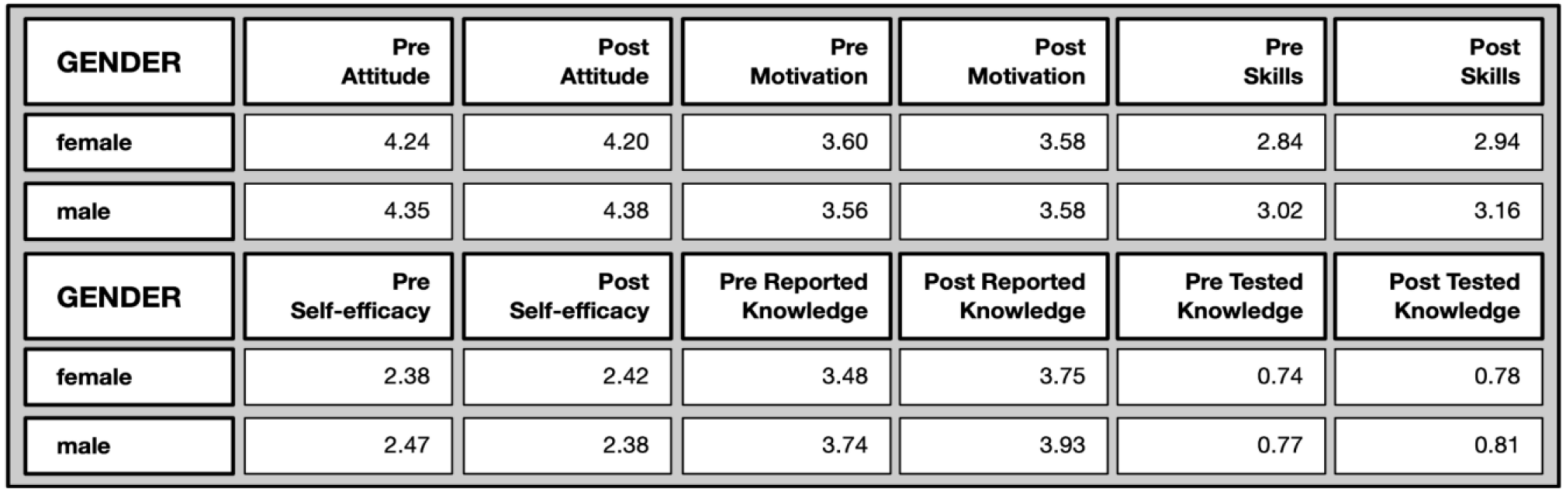
Mean values on different evaluative dimensions by gender.

In the pre- and post-questionnaires there are some, albeit marginal, gender differences when means are compared against each other (cf. Figure 8). These differences, with a few exceptions, were no longer found in the post test. In other words, some of the marginal gender gaps were closed in the post-test with respect to attitudes towards science, as well as tested knowledge, while others persisted (science-related skills and self-reported knowledge).

There is an observed tendency of males having higher scores in attitude towards science before and after participation (pre: U=60403, p=.010; post: U=10497, p=.029), to report higher scientific related skills pre and post (pre: U=60431, p=.007; post: U=10145, p=.036) as well as more scientific knowledge (pre: U= 64639.5, p=.000*; post: U=9721, p=.034). However, when tested for significance with Mann-Whitney U-tests and a corrected Bonferroni-Alpha of 0.000, the only significant difference between the two genders remained for self-reported knowledge before participating in the citizen science projects (marked with a *) and were no longer found in the post test. In other words, some of the marginal gender gaps were closed throughout the participation.

**Figure 9.**
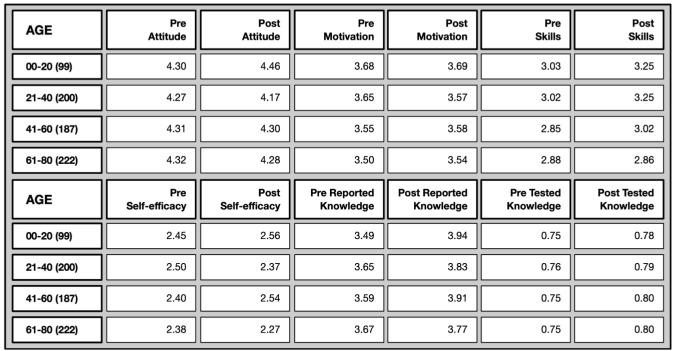
Pre and post mean values on different evaluative dimensions by age groups.

*Age differences* (Overview in Figure 9): Attitude towards science was already high among all age groups in the pre-test and did not change over time. Motivation to join was slightly higher among the youngest age group (compared to the oldest) both in the pre- and post-test. Perceived science-related skills were also greater among younger participants at both time points, while science-related self-efficacy remained consistent across all age groups. While in the pre-test it was the older age group who felt particularly more confident in explaining scientific terms, younger age groups did catch up over time.

**Figure 10.**
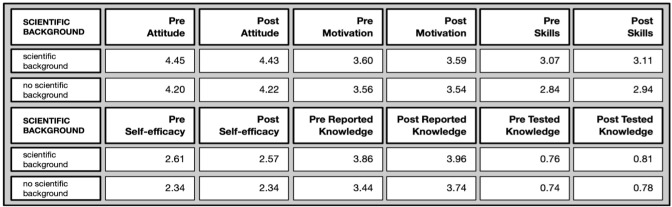
Pre and post comparison of means on different evaluative dimensions by professional background.

*Differences by professional scientific background* (Overview in Figure 10): Most differences appeared between people with scientific backgrounds and people without scientific backgrounds.

In the pre-test, the attitude toward science were more positive/higheramong people with a scientific background (U= 55935, p=.000*), as well as their perceived self-efficacy (U= 54706; p=.000*) and science-related skills (U=50266, p=.001*). However, these differences disappeared in the post-test. Interestingly, there were no differences in the tested knowledge and motivation to join. Marginal differences before participation are closed over time and do not differ significantly between people with and without scientific backgrounds anymore after participating in the citizen science projects.

*Differences by engagement level:* When compared against engagement level (composite indicator of number of categorisations plus total classification time), the only differences appear in the post-questionnaire. Highly engaged people reported more knowledge and a higher motivation to join.

*Differences by visual impairment:* No significant differences resulted from a comparison of people who reported a visual impairment with people who did not.

**Figure 11.**
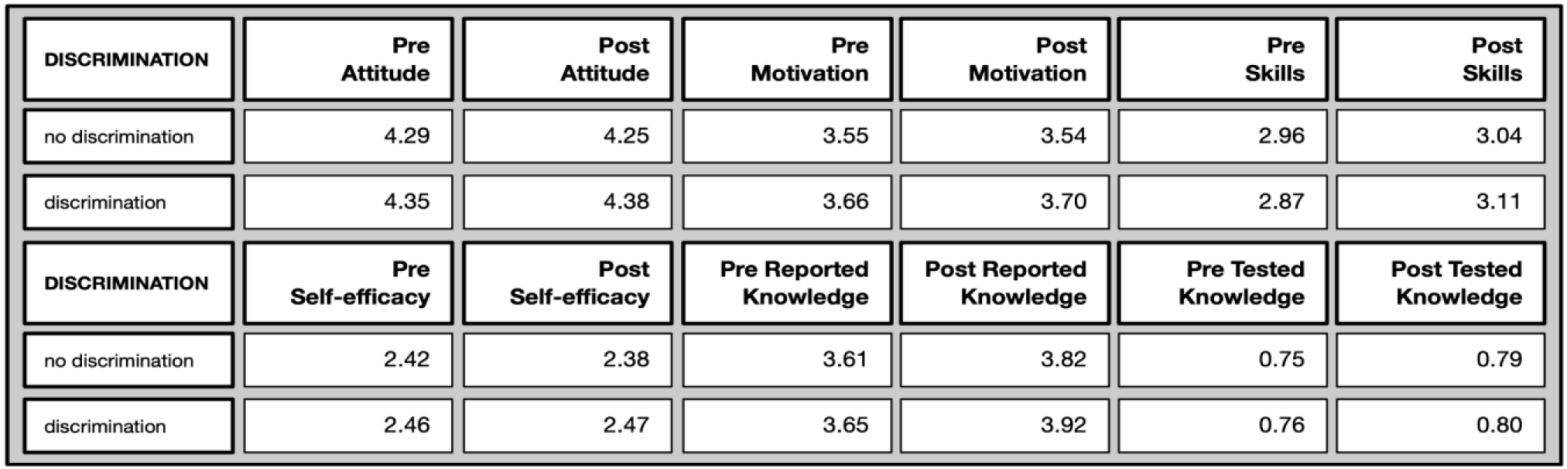
Pre and post mean values on different evaluative dimensions by discrimination experiences.

*Discriminated against group* (Overview in Figure 11): This differentiated based on membership of a discriminated against group (for different reasons, such as gender, sexual orientation, race, migration background, etc), revealing only one marginal but significant difference, as the following overview shows.

The motivation to join was higher for people who felt discriminated against both in the pre (U= 54352, p=.004*) as well as in the post test (U= 9316, p=.014*), We can hypothesise that they valued the option to participate in such a study more because they may otherwise experience fewer opportunities to do so.

## Discussion and conclusion

Citizen science projects face a critical challenge in establishing a unified evaluation standard, hindering effective cross-project comparisons and comprehensive indicators for democratisation in citizen science projects ( Bonney *et al*., 2009; Bonney *et al*., 2014; Jordan *et al*., 2015). Whether citizen science projects are successful in attracting citizens beyond the ‘usual suspects’ is essential to showcase the level of inclusion and accessibility of the project. Our study seeks to address this gap by operationalising the gains of citizen scientists from a democratisation perspective. Furthermore, with our study design we attempt to rectify the limitations of prior studies, such as retrospective analysis, potentially advancing the evolution of evaluation standards in citizen science and to contribute to find out more about people’s motivations and gains per group. However, acknowledging certain drawbacks is essential. The research design's limitations stem from the absence of a control group, making it challenging to attribute observed effects solely to participation. Desirability effects and the self-selection bias of highly motivated participants pose potential concerns. Despite these challenges, our study incorporates objective knowledge measures, bridging the gap between subjective and objective indicators. The divergent procedures for collecting pre- and post-questionnaire data, though unavoidable due to technical constraints as described above, may have influenced post-questionnaire response rates. The procedure for collecting the information in the pre and post questionnaires was not the same. The fact that the latter was accessed via an email link might have resulted in fewer responses in the post-questionnaire as it implies several hurdles (e.g. changed email address, wrong email address, email in junk mail etc.) compared to a link that can be accessed directly within the project that citizen scientists were working on, i.e. the Zooniverse projects.

Considering the results and their limitations, our investigation provides insights into participant benefits and demographic characteristics in citizen science projects.

The first research question reveals positive outcomes, indicating gains in scientific skills and knowledge attributable to participation. While the motivation and attitude toward science remained consistent, our findings align with previous studies, emphasising the transformative potential of citizen science in terms of scientific skill and knowledge gains, even in contributory-focused projects. Obviously, it cannot be ruled out that these gains have been caused by other not-controllable factors. The motivation to join, the attitude towards science and the level of self-efficacy in the science domain does not change over time. This result is similar to other studies ( Bela *et al*., 2016; Richter *et al*., 2016; Riesch & Potter, 2014; Stepenuck & Green, 2015), which also detected knowledge gains and an evolution of scientific skills. It is remarkable that the citizen science projects that are mostly contributory in nature without a deeper involvement of citizen scientists in different phases of the research process, and thus not in line with the extreme citizen science approach, nevertheless led to an increase in the mentioned dimensions. Even a citizen science project with lower levels of involvement as in this study can have positive impacts on knowledge acquisition and the development of scientific skills.

While skills and knowledge improved, other dimensions did not change. The attitude towards science was already high to begin with, not leaving much room for change. On an item-level, we recognise similar motivational drives as have been reported in other studies ( Land-Zandstra *et al*., 2016; Martin *et al*., 2016; Silva *et al*., 2016). People join because they have the desire to contribute to a “greater good,” thus merely for intrinsic rather than extrinsic motivation. The desire to contribute to the objectives of an important or interesting project is an attraction factor for citizens and explains why they join the project in the first place. For this reason, the communication of the project’s mission, achievements and the scientific contributions of the individual citizen science projects is key in recruiting new volunteers and keeping them involved in the project activities.

The fact that the attitude towards science was already positive in the beginning indicates, however, that probably people with negative attitudes towards science are difficult to attract to citizen science projects.

The second research question explores inclusivity and accessibility. Despite a skewed participation pattern towards males with scientific backgrounds and higher education degrees, the projects exhibit inclusivity by engaging participants across genders, ages, and educational backgrounds. Notably, individuals with discrimination experiences are particularly motivated to participate, highlighting the potential of citizen science in empowering marginalised groups. In contrast to the idea of democratisation of access to research and the production of scientific knowledge for all, the participation pattern often to be found in citizen science projects is males with a scientific background and higher educational degrees ( Strasser *et al.*, 2023). Thus, it is important to counteract this pattern of involving solely the ‘usual suspects’ and to analyse the degree of inclusivity and accessibility in citizen science projects. The analysis of the demographic characteristics shows a slight over-representation of males compared to female participants, by more than 10%. Difficulties in attracting women to science studies is a known phenomenon and has to be interpreted in light of general participation in STEM fields (Science Technology Engineering Mathematics) with rates of females differing considerably between countries. Strasser *et al.* (2023), in their meta-analysis of online citizen science projects with more than 14 million participants over two decades, found that “Most citizen science projects, except for nature sensing, are heavily dominated by men, and the vast majority of participants, male and female, have a background in science” ( 2023, p.1). Specifically, they found a male overrepresentation, with 52–90% of participants being male and over 60% employed in the field of science and IT. In another study on nature volunteers ( Ganzevoort & van den Born, 2020), nearly 65% has a higher education degree. Compared to these findings in similar (citizen science) initiatives, the 10% difference between male and female participation, and the 38% of participants reporting a background in science, appear almost negligible. Also participants with lower educational degrees were able to access and contribute to the projects. In terms of age, we find quite a balanced sample, with all age groups represented, from the very young, below the age of 20, to elderly people above the age of 80. Again, compared to the meta-analysis of online citizen science projects by Strasser *et al.* (2023), the age pattern in the four citizen science projects differs from comparable projects, where people in school age account for 35% (against 14% in our case) and elderly above the age of 4% (against 32% of elderly in our case). The participation of elderly people might be attributed to the engagement of the research teams in lectures and courses with the elderly in line with the engagement strategy of the projects. A minority have a background in science, which again indicates that the citizen science projects have successfully attracted people beyond the ‘usual suspects’ ( Curtis, 2018). This is further confirmed by the fact that a considerably high proportion of participants feel that they are members of a discriminated against group and a few indicate a visual impairment speaking also for the technical accessibility of the projects. The demographic analysis of the data reveals that people with discrimination experience are particularly motivated to participate in citizen science projects. Taking into account demographic dimensions, it was not possible to identify any groups of people as having benefited less in terms of knowledge acquisition and development of scientific skills.

Our study suggests that citizen science projects, even with limited citizen involvement, can positively impact knowledge acquisition and scientific skill development. The importance of engagement strategies and accessibility efforts is underscored, emphasising the need for a more diverse participant base, including females, individuals with disabilities, and those with lower educational backgrounds.

Future research should delve into sustained participant impacts, refine evaluation methods, and explore demographic influences further. Emphasising engagement strategies and accessibility testing can foster greater inclusivity, fulfilling the democratising potential of citizen science. Understanding the nuanced gains and influencing factors is crucial for advancing the democratisation of scientific engagement in citizen science projects.

## Ethics and consent

The consent of participants was given online on the Zooniverse platform. For the analysis of the usage data from Zooniverse ( https://www.zooniverse.org/), the REINFORCE participants were informed and protected by the privacy policy statement published on the Zooniverse platform ( https://www.zooniverse.org/privacy), which defines the management and handling of the user data. To register on the platform, the REINFORCE participants had to confirm that they agreed with the policy and they were additionally explicitly asked for their consent allowing their user data collected by Zooniverse to be shared with the REINFORCE project. According to the REINFORCE ethical guidelines, only relevant and necessary data was collected, stored, and analysed.

The Reinforce project underwent an ethics check during grant preparation and as a result dedicated a Work Package to ethics requirements with respective reports and detailed description of ethics processes (POPD, and H Requirement). Additionally, the project provided an ethics handbook comprising a project information sheet, consent sheets, and detailed description of all ethics procedures. The ethics procedures were positively evaluated during project monitoring.

## Data Availability

Zenodo: Pre-Post Questionnaire results of all four demonstrators. https://doi.org/10.5281/zenodo.10728044 ( Unterfrauner, 2024). Zenodo: Pre Survey for Zooniverse GWitchHunters https://doi.org/10.5281/zenodo.11499542 ( Unterfrauner & Fabian, 2024) Data are available under the terms of the Creative Commons Attribution 4.0 International license (CC-BY 4.0).
