# Supplementary material for: What’s in it for citizen scientists? An Analysis of Participant Inclusivity in Citizen Science Projects in Advanced Physics Research
Source: Open Res Eur. 2025 Jan 14;4:124. Originally published 2024 Jun 24. [Version 3] doi: 10.12688/openreseurope.17436.3 (PMC11549546; doi:10.12688/openreseurope.17436.3)
Supplement: Peer review history

# Reviewer report: Reviewer response for version 3

**DOI:** 10.21956/openreseurope.20849.r49741

**Published:** 2025-01-20

**Authors:** Álvaro Padilla-Pozo

**Affiliations:** 1 Sociology, Cornell University, Ithaca, New York, USA

The authors have addressed most of my previous comments. The quality of the manuscript has improved substantively.

If applicable, is the statistical analysis and its interpretation appropriate?

No

Is the study design appropriate and is the work technically sound?

No

Is the work clearly and accurately presented and does it engage with the current literature?

Partly

Are the conclusions drawn adequately supported by the results?

No

Are sufficient details of methods and analysis provided to allow replication by others?

Partly

Are all the source data and materials underlying the results available?

Yes

Reviewer Expertise:

Sociology; Demography; Citizen Science; Statistics

I confirm that I have read this submission and believe that I have an appropriate level of expertise to confirm that it is of an acceptable scientific standard.

# Reviewer report: Reviewer response for version 2

**DOI:** 10.21956/openreseurope.20093.r45161

**Published:** 2024-11-08

**Authors:** Yaqian Wu

**Affiliations:** 1 UCL, London, UK

Thank you for the opportunity to read this paper—it attempts to explore citizen science through the lens of democratisation, which is a timely and important topic. As someone familiar with the field of citizen science but not directly acquainted with your specific research background, I find your approach to democratisation intriguing, with a notable emphasis on inclusivity and representation within citizen science projects. The scope of this paper demonstrates a valuable commitment to addressing some of the broader societal benefits and challenges associated with public engagement in science.

 I have a few suggestions that could further enhance the clarity and impact of your work. I found some points a bit difficult to follow, particularly regarding how the theme of democratisation is structured and linked across different sections.

 In particular, the title and abstract set an expectation for insights on democratisation, yet some sections appear to focus more on general citizen science participation rather than directly examining democratisation, with a stronger emphasis on inclusivity.

 It might be helpful to provide additional specific context on democratisation—such as explicitly conceptualising its meaning within your framework—which could create a more compelling narrative, aiding readers in understanding your perspective on the relationship between inclusivity, representative participation, and democratisation. I recall that during my PhD, I also explored democratisation within the context of citizen science, outlining the relationship between citizen science and scientific democratisation. I began by defining democratisation, highlighting that my study would focus on aspects relevant to scientific democratisation, and then examined the various aspects of citizen science from this angle.

 In your paper, you could consider a similar approach, linking each section back to these definitions, which would clarify how democratisation is represented and assessed within each project, while also preparing readers for the content in the methods section.

 Furthermore, providing a detailed explanation of why the selected case studies (such as astrophysics and particle physics projects) were chosen and how they exemplify your research questions would make the selection criteria clearer to readers.

 In the “Assessing Benefits for Citizen Scientists” section, the discussion begins with project characteristics but does not immediately connect these characteristics to how they benefit citizen scientists themselves within the context of democratisation.

 For a more structured revision, you might consider the following approach:

1. **Clarify and Frame Democratisation: **Start with a strong conceptualisation of democratisation in citizen science, making clear distinctions between inclusion and representation. Consider tying this directly to your research questions to set up a focused exploration.
2. **Strengthen Section Connections: **Ensure each section ties back to the central theme by adding brief summaries that clarify how they contribute to the broader discussion of democratisation.
3. **Case Study Justification: **Explain why specific projects were selected as case studies and how they contribute unique insights into the theme of democratisation.
4. **Results and Analysis Alignment: **Align the results more closely with the elements of democratisation set out in the introduction, particularly to show how data limitations impact findings in this context.

Certainly, you might also consider an alternative approach, as your current paper seems to focus extensively on the theme of “participant inclusivity.” You could consider reframing the paper around a topic like “An Analysis of Participant Inclusivity in Citizen Science Projects in Advanced Physics Research.” This would not only support the argument that these four projects are representative for exploring this subject but would also help readers better understand the participant characteristic data presented in the paper.

 Additionally, the rationale for using the logic model rather than more traditional citizen science evaluation models could be strengthened. For instance, other citizen science evaluation models typically consider not only the assessment of participants but also the scientific validity of data and societal impacts. In contrast, the logic model allows a more focused assessment of participant inclusivity, facilitating a more comprehensive analysis of this aspect. This type of reasoning could be elaborated to reinforce the suitability of the chosen model.

 Some reading materials might help this revision: 

**1) Strasser B et al. (2018 [Ref - 1]) https://infoscience.epfl.ch/entities/publication/07e31c68-e16c-4000-9543-fe47c5ba7859**

**2) Mueller M et al. (2012 [Ref - 2]) https://democracyeducationjournal.org/home/vol20/iss1/2/**

 Overall, I appreciate the insights this paper brings and look forward to seeing how it evolves with these additions!

If applicable, is the statistical analysis and its interpretation appropriate?

Partly

Is the study design appropriate and is the work technically sound?

Partly

Is the work clearly and accurately presented and does it engage with the current literature?

Partly

Are the conclusions drawn adequately supported by the results?

Partly

Are sufficient details of methods and analysis provided to allow replication by others?

Partly

Are all the source data and materials underlying the results available?

Yes

Reviewer Expertise:

I am a researcher specialising in citizen science, with a particular focus on water-related projects that contribute to monitoring Sustainable Development Goal 6 (SDG 6). Since 2018, I have evaluated over 30 such projects across China and the UK. Building on my educational background, my recent research has expanded to include the evaluation of projects related to SDG 15, and I have begun collaborating with CODATA to collect data on citizen science projects across Asia related to the SDGs.

I confirm that I have read this submission and believe that I have an appropriate level of expertise to confirm that it is of an acceptable scientific standard, however I have significant reservations, as outlined above.

## References

[1] : “Citizen Science”? Rethinking Science and Public Participation. *Science & Technology Studies* .2018; 10.23987/sts.60425 52-76 10.23987/sts.60425

[2] : The Future of Citizen Science. *Democracy and Education* .2012;20(1) : Reference source

# Response

**Published:** 2024-12-23

**Authors:** Elisabeth Unterfrauner

**Affiliations:** Technology&Knowledge, Zentrum fur Soziale Innovation, Vienna, Vienna, Austria

**Dear Reviewer,** Thank you very much for your thoughtful and constructive comments, which have greatly contributed to improving the clarity and focus of our paper. In response to your suggestions, we have made several key revisions:

1. **Title Adjustment**: We have adapted the title to reflect a clearer emphasis on inclusiveness and representation, as these are the primary dimensions investigated in our study. As you rightly pointed out, our analysis focuses less on the broader concept of democratisation and more on specific aspects of inclusivity and participant representation. The revised title better aligns with the paper’s scope and content.
2. **Clarification of the Logic Model**: We have now explicitly clarified the rationale for using the logic model and its relevance to our focus on inclusiveness. Specifically, we note that while the logic model also facilitates the measurement of outcomes on the researchers’ side, this aspect is outside the scope of our study. This distinction has been highlighted to ensure readers understand why our study centers on participant-level inclusivity and representation rather than other dimensions of citizen science evaluation.

We appreciate your insights and the references provided, which have helped us refine our approach and improve the alignment between the paper’s content and its framing. Thank you once again for your valuable feedback. We hope the revised manuscript addresses your comments effectively. Kind regards, Elisabeth Unterfrauner

# Response

**Published:** 2025-01-07

**Authors:** Elisabeth Unterfrauner

**Affiliations:** Technology&Knowledge, Zentrum fur Soziale Innovation, Vienna, Vienna, Austria

**Dear Reviewer,** Thank you very much for your thoughtful and constructive comments, which have greatly contributed to improving the clarity and focus of our paper. In response to your suggestions, we have made several key revisions:

1. **Title Adjustment**: We have adapted the title to reflect a clearer emphasis on inclusiveness and representation, as these are the primary dimensions investigated in our study. As you rightly pointed out, our analysis focuses less on the broader concept of democratisation and more on specific aspects of inclusivity and participant representation. The revised title better aligns with the paper’s scope and content.
2. **Clarification of the Logic Model**: We have now explicitly clarified the rationale for using the logic model and its relevance to our focus on inclusiveness. Specifically, we note that while the logic model also facilitates the measurement of outcomes on the researchers’ side, this aspect is outside the scope of our study. This distinction has been highlighted to ensure readers understand why our study centers on participant-level inclusivity and representation rather than other dimensions of citizen science evaluation.

We appreciate your insights and the references provided, which have helped us refine our approach and improve the alignment between the paper’s content and its framing. Thank you once again for your valuable feedback. We hope the revised manuscript addresses your comments effectively. Kind regards,

 Elisabeth Unterfrauner

# Reviewer report: Reviewer response for version 2

**DOI:** 10.21956/openreseurope.20093.r45160

**Published:** 2024-10-21

**Authors:** Álvaro Padilla-Pozo

**Affiliations:** 1 Sociology, Cornell University, Ithaca, New York, USA

This paper attempts to answer two important questions in citizen science: what do citizen scientists gain from participating in citizen science?; and are those gains equally distributed across sociodemographic groups? The paper is well motivated and makes a compelling case about why this is something we should care about if we want science to be inclusive and beneficial for everyone. However, as it stands, the paper suffers from various methodological limitations that restrain the extent to which the authors can answer their research questions rigorously.

 Below, I outline several comments and suggestions that I think the authors should address in future iterations of their manuscript.

 1. There may be a typo in the last paragraph of the introduction. "Our understanding of who volunteers in scientific endeavors are and what motivates them to participate remains limited". I believe the "are" could be mistake

 2. The authors may want to engage with some of the recent literature about demographic and motivational differences in citizen science, e.g. Dekramanjian et al. 2023.

 3. Potential typo. "Whether citizen science initiatives do persist inequalities between more privileged and less privileged people has been investigated in previous studies relying on demographic data of participants, in terms of age, gender, educational background, etc." I believe the authors mean perpetuate and not persist. This sentence would benefit from some clarification.

 4. I believe this sentence needs to be rephrased to improve its clarity. " How to increase the accessibility of citizen science projects, strategies to promote inclusion are being discussed and researched by groups such as the European Citizen Science Association (ECSA) with its working group on Empowerment, inclusiveness, and equity"

 5. Overall, the paper would benefit from explicitly introducing their research questions in the introduction. 

 6. The authors should justify their choice of Zooniverse as a study case. In addition, the authors should discuss the extent to which we may expect the pool of citizen scientists in Zooniverse to be similar or different to other projects, especially since the tasks of their citizen scientists seem to imply a level of difficulty higher than average (compare it to, for example, taking a picture of a constellation, or recording the sound of a bird).

 7. It is misleading to call this a "pre-post" study design. This type of language suggests causality, which is hard to get at with the current structure of the study. First, there is no clear "treatment". For example, some people may have participated more than others (different treatments). Also, we cannot grant that the changes in the measurements of choice were due to taking the treatment or any other reason. Further, there is no "control" group, in fact, everyone is treated. These are all acknowledge by the authors to some extent. 

 The study design is longitudinal, i.e., two waves of data collection where respondents are "followed" over time. This is useful and novel in itself--it has an edge over other studies inquiring into the composition of citizen science projects and the potential heterogeneous distribution of their gains across sociodemographic groups cross-sectionally. I believe the description of the design should change to better reflect its structure.

 8. The methods section needs some structural changes. The discussion about the time frame of the study (beginning "pre" and end "post"), how people were recruited, scales that form each dependent variable, and response rates should all be a part of the methods section and not the results. The methods section should also include the sample size of the first and second wave of data collection, and discuss reasons for a 25.5% response rate. This would help readers to interpret the results from the beginning.

 9. Authors should clarify their "sampling" or recruitment method for this study. Was everyone that signed up to participate in each of the selected Zooniverse projects eligible for this study? What percentage of their participant pool opted into this study?

 10. Similarly, the authors should clarify what "pre" exactly means. Were surveys delivered to people as soon as they signed up to participate in each project?

 11. It is unclear whether the sampling strategy across Zooniverse projects resulted in differences across data quality that the authors should account for. If there were indeed differences, the authors should not merge their data or should model it in a way that accounts for its source (e.g., multivariate models with dummy variables for each project, and joint hypothesis testing). In the results section, the authors state that: "Analyses of participant data showed that there were no significant differences in overall proportion between responses from the four projects. Consequently, the four sets of responses were merged into an overall dataset for further analysis". However, in the discussion section, the authors state that: "The divergent procedures for collecting pre- and post-questionnaire data, though unavoidable due to technical constraints as described above, may have influenced post-questionnaire response rates. The procedure for collecting the information in the pre and post questionnaires was not the same. The fact that the latter was accessed via an email link might have resulted in fewer responses in the post-questionnaire as it implies several hurdles (e.g. changed email address, wrong email address, email in junk mail etc.) compared to a link that can be accessed directly within the project that citizen scientists were working on, i.e. the Zooniverse projects.". This is confusing.

 12. The methods and discussion section would benefit from a thorough examination about how the authors expect selection into the data to impact their results. In the discussion section, the authors state that: "The limitations of the data lie in the fact that, as with all voluntary participation, the dataset is limited to only those people who took the time to complete the questionnaire and thus represents a self-selected sample (Bethlehem, 2010). The data might therefore not be fully representative of all participants in the four projects. " 

 We know that there is "selection" in who decides to participate in citizen science in the first place. We may then think about the people who decided to complete this extra survey as the most "selected" of the pool of citizen scientists. The authors should discuss how one may expect this group to be different from the "average" participant of the projects. That said, I agree with the authors that their sample is nevertheless informative, and their study is important.

 13. It is hard to draw firm conclusions about citizen scientists' gains from participating in these projects given the current study design. The drop in sample size between waves was very substantive (loss of 74.5% of sample), and we do not know if the data for wave two were missing at random--this is very unlikely. The authors need to provide a comparison of the sociodemographic composition of the sample in waves 1 and 2. If wave 2 is substantively different from wave 1, their conclusions would be compromised and the authors should re-weight their data. One efficient way of doing this would be by using inverse probability weighting [2].

 14. The authors say that the survey was filled by 1,179 people, yet, the graphs they include about the age and education distribution of the sample show smaller (and different) sample sizes. This makes the overall sample size of the study unclear. The authors should also have a consistent sample size across their comparisons (they could do a "complete case" analysis).

 15. "On average, the respondents made 109 classifications (with a standard deviation of 826 and a maximum of 24,179 (!!) classifications) and spent 2.09 hours on the project (standard deviation of 14.8 hours and a maximum of 160 hours) ^11^. The average classification time was 60 seconds (with a standard deviation of 112 sec and a max of 595 sec)". 

 These descriptions suggest highly skewed distributions, in which case the median classifications, time spent, and classification time, would be more appropriate measures of centrality of each variable than the mean. 

 16. The authors need to discuss their choice of one month as their time for wave two (this should be a part of the methods section). Should we expect people to substantively improve their knowledge on an area after a month of participating (especially when the authors report that the average time spent on the project was 2.09 hours)?

 17. Authors should discuss whether the significant changes they see are substantive (in terms of size). For example, based on their results, if we were to test someone with a hundred questions about their knowledge on the second wave, we'd expect them to answer two more correct than on the first wave. Is this substantive? Similarly, the changes on the 1-5 scales (skills and reported knowledge) see an improvement of ~.15 points, which would be the equivalent of a .03% increase on a 1-5 scale. What does this all really mean?

 It is surprising (and promising!) that there are any signs of improvement in such a short time window at all (especially given average time of participation). If possible, the authors may want to test a different time window (like a year), if not, they may want to discuss if they expect this trend to continue in the future.

 18. I would suggest reformatting Figures 8 and 9 to improve legibility and comprehension. As they stand, readers must calculate between group differences in wave 1 and wave 2 and assess whether they are substantive. Authors may chose to display, for example, the gender differences in each item instead of the value for men and women. They could have the full table on an appendix.

 19. “In other words, some of the marginal gender gaps were closed in the post-test with respect to attitudes towards science, as well as tested knowledge, while others persisted (science-related skills and self-reported knowledge).”

 Again, it's important that authors discuss which differences are substantive. Statistical significance is not a sign of substantiveness.

If applicable, is the statistical analysis and its interpretation appropriate?

No

Is the study design appropriate and is the work technically sound?

No

Is the work clearly and accurately presented and does it engage with the current literature?

Partly

Are the conclusions drawn adequately supported by the results?

No

Are sufficient details of methods and analysis provided to allow replication by others?

Partly

Are all the source data and materials underlying the results available?

Yes

Reviewer Expertise:

Sociology; Demography; Citizen Science; Statistics

I confirm that I have read this submission and believe that I have an appropriate level of expertise to state that I do not consider it to be of an acceptable scientific standard, for reasons outlined above.

## References

[1] : Demographic and motivational differences between participants in analog and digital citizen science projects for monitoring mosquitoes. *Sci Rep* .2023;13(1) : 10.1038/s41598-023-38656-y 12384 10.1038/s41598-023-38656-y 37524770 PMC10390545

[2] : Review of inverse probability weighting for dealing with missing data. *Stat Methods Med Res* .2013;22(3) : 10.1177/0962280210395740 278-95 10.1177/0962280210395740 21220355

# Response

**Published:** 2024-12-23

**Authors:** Elisabeth Unterfrauner

**Affiliations:** Technology&Knowledge, Zentrum fur Soziale Innovation, Vienna, Vienna, Austria

Dear reviewer, Thank you very much for your thoughtful recommendations and suggestions to improve the article. We greatly appreciate the time and effort you have invested in reviewing our work. In the following, we provide a detailed response to each of your comments, addressing them in the same order as presented in your review. Most of the issues raised have been addressed in the revised manuscript. However, there are some instances where we respectfully disagree or where the points raised were already addressed in the version you reviewed. These have been clarified in our responses below. Thank you again for your constructive feedback, which has helped us enhance the quality of the manuscript and to revise the paper in a next version. Kind regards,

 Elisabeth Unterfrauner  

1. We have corrected the typo.
2. We have added a reference to Dekramanjian et al. (2023) in the section regarding motivations to participate in the introduction.
3. Typo has been corrected.  
4. We have rephrased the sentence.
5. We have introduced the research questions earlier as suggested and have added information on the structure of the paper to enhance the logical flow.
6. Thank you for highlighting this point. We have now made our justification for choosing Zooniverse as a study case more explicit by referencing the REINFORCE project and its accompanying evaluation work, which specifically focused on engaging citizen scientists in scientific projects hosted on this platform. Additionally, we have included a discussion on how the pool of citizen scientists in Zooniverse might compare to those in other citizen science projects.
7. While we appreciate your concerns about the use of "pre-post" terminology and its implications for causality, we believe that our description of the study as a “one-group pre-test/post-test design” is accurate within the framework of quasi-experimental research.

In the **Methods and Materials** section, we explicitly define and discuss the limitations of this design, stating:

*"To measure the outcomes at the individual participant level, a one-group pre-test/post-test design (Levine & Parkinson, 2014) was implemented. This design is classified as quasi-experimental because it lacks two key components of true experimental designs: a control or comparison group, and the random selection and assignment of participants. As a result, while it is possible to infer that changes observed between the pre-test and post-test are associated with participants’ involvement in citizen science projects, it cannot be definitively concluded that these changes were solely caused by such participation. External factors cannot be ruled out or controlled for in this design."* We agree that the absence of a control group and the variability in participation levels among respondents are important limitations, and these have been transparently addressed in the manuscript. Furthermore, we acknowledge that "pre-post" designs can imply causality, but in this context, we use the term strictly to describe the temporal structure of data collection rather than to assert causal relationships. Finally, we appreciate the reviewer’s recognition of the longitudinal nature of our design, as well as its novelty and value in comparison to purely cross-sectional approaches.

1. In response, we have made several revisions to improve clarity and ensure that relevant details are included in the appropriate sections:
**Details on the Sample:** Information about the sample size for both the first and second waves of data collection has been moved to the methods section, along with additional context to help readers better interpret the results.
**Participant Recruitment:** A brief description of how participants were recruited has been added to provide a clearer understanding of the recruitment process and its potential implications for the study.
**Response Rate Discussion:** We have included a short discussion addressing the 25.5% response rate in the second round, acknowledging potential reasons for this lower rate and its implications for the study's findings.
**Timeframe of Data Collection:** The timeframe of data collection was already included in the methods section, but we have revisited and clarified this information to ensure it is prominently placed and easy to locate.
2. Details on recruitment have been added.
3. We believe this is already addressed in the manuscript, but we have clarified it further to ensure readers fully understand what "pre" means in the context of our study. As described in the paper:

*"The link to the pre-questionnaire was included in the Zooniverse project tutorials. These tutorials were a required step for participants to understand how to classify data and contribute to individual projects. Participants were invited to complete the pre-questionnaire during this stage, prior to beginning their contributions to the project. They were also asked to share their email addresses upon completing the pre-questionnaire to facilitate follow-up. One month after completing the pre-questionnaire, participants received an email with a link to the post-questionnaire."* This process ensures that the "pre" phase refers to participants' initial stage of engagement with the project, before they began their active participation.

1. The divergent procedure for collecting the responses to the post-questionnaire refers to a difference in the data collection method between the pre- and post-questionnaires, not between the four projects. Importantly, the procedure for both pre- and post-questionnaire data collection was identical across all four citizen science projects included in this study. To clarify this, we have made this explicit in the manuscript.

As described in the paper: *" The link to the pre-questionnaire was included in the four Zooniverse project tutorials. These tutorials were a required step for participants to understand how to classify data and contribute to individual projects. Participants were invited to complete the pre-questionnaire during this stage, prior to beginning their contributions to the project. They were also asked to share their email addresses upon completing the pre-questionnaire to facilitate follow-up.." and "* *While the pre-questionnaire was filled in by a total of 1,179 participants, after data cleaning for incomplete responses, the sample was reduced to 737 responses. The post-questionnaire received 301 responses, resulting in an overall response rate of 25.5% and approximately 42% relative to the cleaned data sample. The limited response rate in the second round can likely be attributed to technical constraints, as responses were collected via email rather than directly through the Zooniverse portal, as was the case for the first round. "* We acknowledge that this procedural difference may have introduced hurdles for participants in completing the post-questionnaire (e.g., invalid email addresses, emails being flagged as junk mail, or participants no longer using the email they provided), and this is reflected in the lower response rate. However, this procedure was consistently applied across all four projects, and no project-specific differences were introduced as a result. Furthermore, as noted in the results section, our analyses showed no significant differences in the overall proportions of responses across the four projects. Based on this finding, we merged the datasets for analysis, as any differences in response characteristics were not statistically significant. To address potential confusion, we have now explicitly clarified in the manuscript that the procedures and datasets were consistent across all four projects. We hope this clarification resolves any concerns regarding the sampling strategy and the rationale for merging the datasets.

1. We have added hypotheses on the self-selection biases of respondents in two sections of the paper (sampling and discussion).
2. We acknowledge the concern regarding the drop in sample size between waves and its potential implications. However, the core of our analysis focuses on a pre-post comparison of the same individuals, rather than a comparison between separate participant groups from wave 1 and wave 2. Specifically, the research questions concerning participant gains are addressed by analysing responses from participants who completed both the pre- and post-questionnaires. This approach ensures that demographic differences between waves do not influence the results, as the same individuals are compared across the two time points.

The statistical analysis employed—the Wilcoxon signed-rank test—is designed for paired samples and is appropriate for this one-group pre-test/post-test design. This method inherently controls for individual demographic variables by focusing solely on within-participant changes over time. Whilst we recognise the value of re-weighting in studies comparing distinct samples, it is not applicable in this context, as our analysis does not compare wave 1 with wave 2 data but instead examines changes within the conjunct sample of participants who completed both waves.

1. We have made the necessary adaptation.
2. We have added the medians and agree that is a skewed sample towards power users.
3. We believe this is already addressed in the manuscript, but we appreciate the opportunity to clarify further. As noted in the version you reviewed: *"The timeframe for sending out the post-questionnaire after one month was chosen as appropriate because experiences from other Zooniverse projects suggested that most participant classifications are completed within this period. Additionally, other studies in the field have utilised a similar timeframe to measure active engagement and outcomes in citizen science projects (Strasser et al., 2023)." *This timeframe was deemed reasonable as it captures participant engagement and learning outcomes during or shortly after the active participation period, which is typically concentrated within the first month. Although the average time spent on the project (2.09 hours) may appear short, this reflects the cumulative classification work participants undertake, often spread across multiple short sessions. We hope this explanation helps clarify our rationale for selecting the one-month period, and we have ensured this justification is prominently included in the **Methods** section for greater visibility.
4.  Effect sizes have been added to estimate the significance of change.
5. Thank you for your suggestion to reformat Figures 8 and 9 to improve their legibility and comprehension. While we understand your recommendation to display only the differences between groups (e.g., gender differences for each item), we decided not to include a table showing only the differences. Such a table would not provide information on the direction of the differences, which we believe is critical for a complete understanding of the results.

To ensure clarity and maintain the ability for readers to interpret both the magnitude and direction of the differences, we have opted to retain the current format of Figures 8 and 9, which display the values for each group separately. We believe this approach provides a more comprehensive visualisation of the data, allowing readers to assess group differences while still understanding the underlying values.

1. To address this, we have now included effect sizes in our analysis. By reporting effect sizes alongside statistical significance, we provide a clearer indication of the magnitude of the observed differences, enabling readers to better assess their practical and substantive relevance. We believe this addition enhances the interpretability of our findings and aligns with your recommendation to go beyond statistical significance when discussing the results.

# Response

**Published:** 2025-01-07

**Authors:** Elisabeth Unterfrauner

**Affiliations:** Technology&Knowledge, Zentrum fur Soziale Innovation, Vienna, Vienna, Austria

Dear reviewer, Thank you very much for your thoughtful recommendations and suggestions to improve the article. We greatly appreciate the time and effort you have invested in reviewing our work. In the following, we provide a detailed response to each of your comments, addressing them in the same order as presented in your review. Most of the issues raised have been addressed in the revised manuscript. However, there are some instances where we respectfully disagree or where the points raised were already addressed in the version you reviewed. These have been clarified in our responses below. Thank you again for your constructive feedback, which has helped us enhance the quality of the manuscript and to revise the paper in a next version.   Kind regards,

 Elisabeth Unterfrauner  

1. We have corrected the typo.
2. We have added a reference to Dekramanjian et al. (2023) in the section regarding motivations to participate in the introduction.
3. Typo has been corrected.  
4. We have rephrased the sentence.
5. We have introduced the research questions earlier as suggested and have added information on the structure of the paper to enhance the logical flow.
6. Thank you for highlighting this point. We have now made our justification for choosing Zooniverse as a study case more explicit by referencing the REINFORCE project and its accompanying evaluation work, which specifically focused on engaging citizen scientists in scientific projects hosted on this platform. Additionally, we have included a discussion on how the pool of citizen scientists in Zooniverse might compare to those in other citizen science projects.
7. While we appreciate your concerns about the use of "pre-post" terminology and its implications for causality, we believe that our description of the study as a “one-group pre-test/post-test design” is accurate within the framework of quasi-experimental research.

In the **Methods and Materials** section, we explicitly define and discuss the limitations of this design, stating:

*"To measure the outcomes at the individual participant level, a one-group pre-test/post-test design (Levine & Parkinson, 2014) was implemented. This design is classified as quasi-experimental because it lacks two key components of true experimental designs: a control or comparison group, and the random selection and assignment of participants. As a result, while it is possible to infer that changes observed between the pre-test and post-test are associated with participants’ involvement in citizen science projects, it cannot be definitively concluded that these changes were solely caused by such participation. External factors cannot be ruled out or controlled for in this design."* We agree that the absence of a control group and the variability in participation levels among respondents are important limitations, and these have been transparently addressed in the manuscript. Furthermore, we acknowledge that "pre-post" designs can imply causality, but in this context, we use the term strictly to describe the temporal structure of data collection rather than to assert causal relationships. Finally, we appreciate the reviewer’s recognition of the longitudinal nature of our design, as well as its novelty and value in comparison to purely cross-sectional approaches.

1. In response, we have made several revisions to improve clarity and ensure that relevant details are included in the appropriate sections: **Details on the Sample:** Information about the sample size for both the first and second waves of data collection has been moved to the methods section, along with additional context to help readers better interpret the results. **Participant Recruitment:** A brief description of how participants were recruited has been added to provide a clearer understanding of the recruitment process and its potential implications for the study. **Response Rate Discussion:** We have included a short discussion addressing the 25.5% response rate in the second round, acknowledging potential reasons for this lower rate and its implications for the study's findings. **Timeframe of Data Collection:** The timeframe of data collection was already included in the methods section, but we have revisited and clarified this information to ensure it is prominently placed and easy to locate.
2. Details on recruitment have been added.
3. We believe this is already addressed in the manuscript, but we have clarified it further to ensure readers fully understand what "pre" means in the context of our study. As described in the paper:

*"The link to the pre-questionnaire was included in the Zooniverse project tutorials. These tutorials were a required step for participants to understand how to classify data and contribute to individual projects. Participants were invited to complete the pre-questionnaire during this stage, prior to beginning their contributions to the project. They were also asked to share their email addresses upon completing the pre-questionnaire to facilitate follow-up. One month after completing the pre-questionnaire, participants received an email with a link to the post-questionnaire."* This process ensures that the "pre" phase refers to participants' initial stage of engagement with the project, before they began their active participation.

1. The divergent procedure for collecting the responses to the post-questionnaire refers to a difference in the data collection method between the pre- and post-questionnaires, not between the four projects. Importantly, the procedure for both pre- and post-questionnaire data collection was identical across all four citizen science projects included in this study. To clarify this, we have made this explicit in the manuscript.

As described in the paper: *" The link to the pre-questionnaire was included in the four Zooniverse project tutorials. These tutorials were a required step for participants to understand how to classify data and contribute to individual projects. Participants were invited to complete the pre-questionnaire during this stage, prior to beginning their contributions to the project. They were also asked to share their email addresses upon completing the pre-questionnaire to facilitate follow-up.." and "* *While the pre-questionnaire was filled in by a total of 1,179 participants, after data cleaning for incomplete responses, the sample was reduced to 737 responses. The post-questionnaire received 301 responses, resulting in an overall response rate of 25.5% and approximately 42% relative to the cleaned data sample. The limited response rate in the second round can likely be attributed to technical constraints, as responses were collected via email rather than directly through the Zooniverse portal, as was the case for the first round. "* We acknowledge that this procedural difference may have introduced hurdles for participants in completing the post-questionnaire (e.g., invalid email addresses, emails being flagged as junk mail, or participants no longer using the email they provided), and this is reflected in the lower response rate. However, this procedure was consistently applied across all four projects, and no project-specific differences were introduced as a result. Furthermore, as noted in the results section, our analyses showed no significant differences in the overall proportions of responses across the four projects. Based on this finding, we merged the datasets for analysis, as any differences in response characteristics were not statistically significant. To address potential confusion, we have now explicitly clarified in the manuscript that the procedures and datasets were consistent across all four projects. We hope this clarification resolves any concerns regarding the sampling strategy and the rationale for merging the datasets.

1. We have added hypotheses on the self-selection biases of respondents in two sections of the paper (sampling and discussion).
2. We acknowledge the concern regarding the drop in sample size between waves and its potential implications. However, the core of our analysis focuses on a pre-post comparison of the same individuals, rather than a comparison between separate participant groups from wave 1 and wave 2. Specifically, the research questions concerning participant gains are addressed by analysing responses from participants who completed both the pre- and post-questionnaires. This approach ensures that demographic differences between waves do not influence the results, as the same individuals are compared across the two time points.

The statistical analysis employed—the Wilcoxon signed-rank test—is designed for paired samples and is appropriate for this one-group pre-test/post-test design. This method inherently controls for individual demographic variables by focusing solely on within-participant changes over time. Whilst we recognise the value of re-weighting in studies comparing distinct samples, it is not applicable in this context, as our analysis does not compare wave 1 with wave 2 data but instead examines changes within the conjunct sample of participants who completed both waves.

1. We have made the necessary adaptation.
2. We have added the medians and agree that is a skewed sample towards power users.
3. We believe this is already addressed in the manuscript, but we appreciate the opportunity to clarify further. As noted in the version you reviewed: *"The timeframe for sending out the post-questionnaire after one month was chosen as appropriate because experiences from other Zooniverse projects suggested that most participant classifications are completed within this period. Additionally, other studies in the field have utilised a similar timeframe to measure active engagement and outcomes in citizen science projects (Strasser et al., 2023)." *This timeframe was deemed reasonable as it captures participant engagement and learning outcomes during or shortly after the active participation period, which is typically concentrated within the first month. Although the average time spent on the project (2.09 hours) may appear short, this reflects the cumulative classification work participants undertake, often spread across multiple short sessions. We hope this explanation helps clarify our rationale for selecting the one-month period, and we have ensured this justification is prominently included in the **Methods** section for greater visibility.
4.  Effect sizes have been added to estimate the significance of change.
5. Thank you for your suggestion to reformat Figures 8 and 9 to improve their legibility and comprehension. While we understand your recommendation to display only the differences between groups (e.g., gender differences for each item), we decided not to include a table showing only the differences. Such a table would not provide information on the direction of the differences, which we believe is critical for a complete understanding of the results.

To ensure clarity and maintain the ability for readers to interpret both the magnitude and direction of the differences, we have opted to retain the current format of Figures 8 and 9, which display the values for each group separately. We believe this approach provides a more comprehensive visualisation of the data, allowing readers to assess group differences while still understanding the underlying values.

1. To address this, we have now included effect sizes in our analysis. By reporting effect sizes alongside statistical significance, we provide a clearer indication of the magnitude of the observed differences, enabling readers to better assess their practical and substantive relevance. We believe this addition enhances the interpretability of our findings and aligns with your recommendation to go beyond statistical significance when discussing the results.

# Reviewer report: Reviewer response for version 1

**DOI:** 10.21956/openreseurope.18843.r41748

**Published:** 2024-08-05

**Authors:** Laura Verbrugge

**Affiliations:** 1 Aalto University, Espoo, Finland

This paper evaluates individual gains of participating in four citizen science (CS) projects where participants performed categorization of astrophysics and particle physics data on the Zooniverse platform. The four CS projects were newly created as part of the REINFORCE EU project and are particularly praiseworthy for their inclusion strategy focusing on elderly people and people with visual impairments. I have not come across such inclusive citizen science projects in the scientific literature before and therefore this paper can make an important contribution.

 My overall assessment is that this paper has more potential that can be tapped into, in particular with regards to the inclusion element. Also, some clarifications are needed on the part of Research Question 2 and statistical analyses that were performed. I elaborate on this in more detail in my comments below.

 The introduction of the paper is informative and cites recent and relevant literature. However, it is a very general introduction to CS that could be tailored more to the actual novel element in the study, i.e. focusing on inclusion and representation. In addition to the current content (on extreme citizen science and bias in online CS), the authors could cite more papers on representation and bias of certain groups in different citizen science projects (e.g. Paleco et al. 2021 [Ref - 3]; Strasser et al. 2023 [Ref - 2]). It could also be worth expanding beyond online CS project to for example biodiversity monitoring (e.g. Ganzevoort et al. 2020 [Ref - 1]) and other fields. If the focus is on online participation, it would be helpful to give an account of what could be common or persistent handicaps or barriers towards participation (which could explain why e.g. elderly or visually impaired people do not participate as often?).

 In my opinion, research question 2 requires some more attention as the current formulation is not clear. Can you reformulate to make it more specific and measurable?

 As a related comment, I got very interested in the inclusion strategies that you implemented in the project. And I would like to have read more about the effectiveness of the strategies in recruiting new/other participants compared to similar projects. Do you have any data that could provide insights into this, and if so, would it be worth adding it? For example, do you know how many of the course and workshop participants actually signed up to the CS project? Are there any other studies that have targeted elderly and visually impaired people that you could compare with? How was the approach of using sound received by the scientists that were analysing data? I understand that perhaps some of these questions go beyond the scope of the current study, however, you could consider to what extent they could be integrated.

 Overall, the methods are well described and nicely supported by Figures. I recommend to replace the Figures 2-5 (with screenshots) with one Table that gives information about each CS project, including name, goals, type (e.g. contributory, collaborative), no of participants, url etc. The current Figures 2-5 do not have added value in my opinion and could be removed.

 Regarding the statistical analyses, I was missing a clear description of the type of SPSS tests that were used for the comparison (before-after and between groups) to establish whether the differences are significant and whether the data was suitable for performing these tests (met the assumptions).

 Also, while Figure 10 provides an overview of the comparison stats, Figures 11 -14 do not and these should be added to support the conclusions drawn.

 The limitations of the study are clearly described. In addition to the current content, the authors could be encouraged to reflect more on some methodological choices, e.g. to measure the effects after one month of participation (is this sufficient time to measure these type of effects?) and reasons for outliers and different levels of engagement among participants while engaging with other literature.

 To conclude, this study is very promising and further improvements as suggested will improve the quality and novelty of the paper.

If applicable, is the statistical analysis and its interpretation appropriate?

Partly

Is the study design appropriate and is the work technically sound?

Yes

Is the work clearly and accurately presented and does it engage with the current literature?

Partly

Are the conclusions drawn adequately supported by the results?

Yes

Are sufficient details of methods and analysis provided to allow replication by others?

Partly

Are all the source data and materials underlying the results available?

Yes

Reviewer Expertise:

citizen science, stakeholder engagement, quantitative methods, motivations

I confirm that I have read this submission and believe that I have an appropriate level of expertise to confirm that it is of an acceptable scientific standard, however I have significant reservations, as outlined above.

## References

[1] : Understanding citizens’ action for nature: The profile, motivations and experiences of Dutch nature volunteers. *Journal for Nature Conservation* .2020;55: 10.1016/j.jnc.2020.125824 10.1016/j.jnc.2020.125824

[2] : Quantifying online citizen science: Dynamics and demographics of public participation in science. *PLoS One* .2023;18(11) : 10.1371/journal.pone.0293289 e0293289 10.1371/journal.pone.0293289 37988360 PMC10662724

[3] : Inclusiveness and Diversity in Citizen Science. *The Science of Citizen Science. Springer, Cham.* .2021;261-281 Reference source

# Response

**Published:** 2024-09-20

**Authors:** Elisabeth Unterfrauner

**Affiliations:** Technology&Knowledge, Zentrum fur Soziale Innovation, Vienna, Vienna, Austria

Dear reviewer, Thank you for your thorough review and very helpful suggestions! We have addressed all your comments and believe that the revisions have significantly improved the quality of the paper.

 We have expanded the section on the inclusivity aspects of the citizen science initiatives, emphasising the uniqueness of the paper, which you highlighted: *“I have not come across such inclusive citizen science projects in the scientific literature before and therefore this paper can make an important contribution.”*

 Please find below our answers to the suggestions:

**(1) Introduction:**

***Reviewer*** ***Comment: **“The introduction of the paper is informative and cites recent and relevant literature. However, it is a very general introduction to CS that could be tailored more to the actual novel element in the study, i.e. focusing on inclusion and representation. In addition to the current content (on extreme citizen science and bias in online CS), the authors could cite more papers on representation and bias of certain groups in different citizen science projects (e.g. Paleco et al. 2021 [Ref - 3]; Strasser et al. 2023 [Ref - 2]). It could also be worth expanding beyond online CS project to for example biodiversity monitoring (e.g. Ganzevoort et al. 2020 [Ref - 1]) and other fields. If the focus is on online participation, it would be helpful to give an account of what could be common or persistent handicaps or barriers towards participation (which could explain why e.g. elderly or visually impaired people do not participate as often?).”*

**Author Response:** Thank you for the helpful references. We have added citations to the recommended literature and additionally references regarding bias and representation of various groups in citizen science projects and beyond. We have also included a paragraph on the „Accessibility and inclusiveness of citizen science projects” and compared our demographic results with similar studies to highlight the accessibility and inclusiveness of the four citizen science projects discussed in the paper.

 (2) **Research question 2:**

***Reviewer*** ***Comment:** “In my opinion, research question 2 requires some more attention as the current formulation is not clear. Can you reformulate to make it more specific and measurable?”*

**Author Response:**  We have refined research question 2 to make it clearer and more operationalisable.

 (3) **Inclusion strategies:**

***Reviewer*** ***Comment:** “As a related comment, I got very interested in the inclusion strategies that you implemented in the project. And I would like to have read more about the effectiveness of the strategies in recruiting new/other participants compared to similar projects. Do you have any data that could provide insights into this, and if so, would it be worth adding it? For example, do you know how many of the course and workshop participants actually signed up to the CS project? Are there any other* *studies that have targeted elderly and visually impaired people that you could compare with? How was the approach of using sound received by the scientists that were analysing data? I understand that perhaps some of these questions go beyond the scope of the current study, however, you could consider to what extent they could be integrated.”*

**Author Response:** We have added more details regarding the inclusion strategies for elderly and visually impaired individuals, including results from sessions and data quality assessments. While we did collect some information in the pre-post questionnaires regarding participants’ attendance at events, the responses were limited and not reliable enough to provide a sound estimate of how many participants joined the Zooniverse projects after attending events.

 (4) **Figures:**

***Reviewer*** ***Comment:** “Overall, the methods are well described and nicely supported by Figures. I recommend to replace the Figures 2-5 (with screenshots) with one Table that gives information about each CS project, including name, goals, type (e.g. contributory, collaborative), no of participants, url etc. The current Figures 2-5 do not have added value in my opinion and could be removed.”*

**Author Response:** We have removed Figures 2-5 and replaced them with a table summarising the relevant information about each CS project.

 (5) **More information on statistical tests:**

***Reviewer*** ***Comment:** “Regarding the statistical analyses, I was missing a clear description of the type of SPSS tests that were used for the comparison (before-after and between groups) to establish whether the differences are significant and whether the data was suitable for performing these tests (met the assumptions). Also, while Figure 10 provides an overview of the comparison stats, Figures 11 -14 do not and these should be added to support the conclusions drawn.”*

**Author Response:** We have replaced the paired-sample t-tests with non-parametric Wilcoxon and Mann-Whitney-U tests and have added a description on these statistical tests. While the differences remain the same, some differences are less pronounced resulting in fewer signifcant differences. The figures have been updated, and the new statistical parameters are now either included in the figures themselves or the accompanying text.

 (6) **Reflection on methodological choice:**

***Reviewer*** ***Comment:** “The limitations of the study are clearly described. In addition to the current content, the authors could be encouraged to reflect more on some methodological choices, e.g. to measure the effects after one month of participation (is this sufficient time to measure these type of effects?) and reasons for outliers and different levels of engagement among participants while engaging with other literature.”*

**Author Response: **We have added a brief paragraph in the *“Methods and Materials”* section explaining the rationale behind the chosen timeframe. The research questions are focused on gains in various areas, not the stability of these gains, which is why we opted for a pre-post design with measurements taken shortly after the last engagement with the Zooniverse platform. Similar timeframes have been used in related studies and were recommended by colleagues from the Zooniverse team.  

 We hope these revisions meet your expectations and address all your concerns. We greatly appreciate your valuable feedback, which has strengthened our manuscript. Should you have any further suggestions or questions, we would be happy to address them. Thank you once again for your time and consideration.

 Sincerely, Elisabeth Unterfrauner and colleagues

# Reviewer report: Reviewer response for version 1

**DOI:** 10.21956/openreseurope.18843.r41742

**Published:** 2024-07-25

**Authors:** Alessio Livio Spera

**Affiliations:** 1 Agenzia Per La Promozione Della Ricerca Europea, Rome, Italy

It's impressive the way this study "flips" the common perspective of Citizen Science as a disruptively inclusive methodology and focuses on its challenge to face diversities instead, and actually investigates its difficulties and concrete results in terms of unification and democratisation potential. 

 Some of the highlighted findings are extremely powerful for what they represent and what they could potentially mobilise, such as: the fact that "people who feel that they belong to a discriminated group are particularly motivated to participate in citizen science projects", or the fact that more than half participants indicated that they had no professional background in science, or finally that some of the marginal gender gaps were closed in the post-test with respect to attitudes towards science.

 On a side, yet relevant, note, the introduction provide a smooth transition from the background definition to the real topic of the article, which is an enormous added value for the reader.

 Also, the explanation of the baseline methodology is clear, well explained, and convincing, with a high level of details richness, for example in the breakdown into 10 steps of the questionnaire construction process, which is more than useful. Nonetheless, solid considerations are provided about the desk research on citizen science studies, thus giving credit to the uniqueness of this work, and the bibliographic background is strong and clear.

 All the 14 Figures actually help reading the data and findings, representing a significant contribution to the appreciation of the article.

 Overall, the research article is interesting and able to make the curiosity of the reader grow throughout the pages, which is a rare feature in this kind of works. Most of the findings are brilliant, some of them are even inspiring, which is a plus. It is definitely a great piece of work for those who are in the field, to get to know more about Citizen Science and its less addressed implications.

If applicable, is the statistical analysis and its interpretation appropriate?

Yes

Is the study design appropriate and is the work technically sound?

Yes

Is the work clearly and accurately presented and does it engage with the current literature?

Yes

Are the conclusions drawn adequately supported by the results?

Yes

Are sufficient details of methods and analysis provided to allow replication by others?

Yes

Are all the source data and materials underlying the results available?

Yes

Reviewer Expertise:

Project Management, Citizen Science, Science and Society, Stakeholder Engagement

I confirm that I have read this submission and believe that I have an appropriate level of expertise to confirm that it is of an acceptable scientific standard.

# Response

**Published:** 2024-09-20

**Authors:** Elisabeth Unterfrauner

**Affiliations:** Technology&Knowledge, Zentrum fur Soziale Innovation, Vienna, Vienna, Austria

Dear Reviewer, Thank you for your thoughtful and encouraging feedback. We are truly grateful for your recognition of the study’s focus on the challenges of citizen science in fostering inclusivity and democratization, which we aimed to highlight as a critical area of exploration. We are especially pleased that the findings regarding participant motivations and the closing of marginal gender gaps resonated with you, as these represent key insights from our research. We sincerely appreciate your positive and insightful comments, which inspire us to continue exploring these important questions in citizen science. Best regards,

 Elisabeth Unterfrauner
